# *De novo* assembly and characterization of transcriptome using Illumina paired-end sequencing and identification of CesA gene in ramie (*Boehmeria nivea L. Gaud*)

**DOI:** 10.1186/1471-2164-14-125

**Published:** 2013-02-26

**Authors:** Touming Liu, Siyuan Zhu, Qingming Tang, Ping Chen, Yongting Yu, Shouwei Tang

**Affiliations:** 1 Institute of Bast Fiber Crops and Center of Southern Economic Crops, Chinese Academy of Agricultural Sciences, Changsha 410205, China

## Abstract

**Background:**

Ramie fiber, extracted from vegetative organ stem bast, is one of the most important natural fibers. Understanding the molecular mechanisms of the vegetative growth of the ramie and the formation and development of bast fiber is essential for improving the yield and quality of the ramie fiber. However, only 418 expressed tag sequences (ESTs) of ramie deposited in public databases are far from sufficient to understand the molecular mechanisms. Thus, high-throughput transcriptome sequencing is essential to generate enormous ramie transcript sequences for the purpose of gene discovery, especially genes such as the cellulose synthase (CesA) gene.

**Results:**

Using Illumina paired-end sequencing, about 53 million sequencing reads were generated. *De novo* assembly yielded 43,990 unigenes with an average length of 824 bp. By sequence similarity searching for known proteins, a total of 34,192 (77.7%) genes were annotated for their function. Out of these annotated unigenes, 16,050 and 13,042 unigenes were assigned to gene ontology and clusters of orthologous group, respectively. Searching against the Kyoto Encyclopedia of Genes and Genomes Pathway database (KEGG) indicated that 19,846 unigenes were mapped to 126 KEGG pathways, and 565 genes were assigned to http://starch and sucrose metabolic pathway which was related with cellulose biosynthesis. Additionally, 51 CesA genes involved in cellulose biosynthesis were identified. Analysis of tissue-specific expression pattern of the 51 CesA genes revealed that there were 36 genes with a relatively high expression levels in the stem bark, which suggests that they are most likely responsible for the biosynthesis of bast fiber.

**Conclusion:**

To the best of our knowledge, this study is the first to characterize the ramie transcriptome and the substantial amount of transcripts obtained will accelerate the understanding of the ramie vegetative growth and development mechanism. Moreover, discovery of the 36 CesA genes with relatively high expression levels in the stem bark will present an opportunity to understand the ramie bast fiber formation and development mechanisms.

## Background

Sequencing and analysis of expressed sequence tags (ESTs) has been a primary tool in gene discovery and genomic sequence annotation in plants. Additionally, ESTs can be used for other functional genomic projects, including gene expression profiling, microarrays, molecular markers, and physical mapping. Over the past ten years, in order to obtain the transcribe EST information, a large number of cDNA libraries have been constructed and sequenced for rice [1], maize [2,3], wheat [4] and other crops. However, traditional sequencing methods used for the generation of ESTs require costly and time-consuming approaches involving cDNA library construction, cloning, and the labor-intensive Sanger sequencing. Alternatively, the transcriptome analysis based on the next generation sequencing (NGS) is more attractive in identifying the expression gene for its characteristics of cost-efficient, high throughput and rapidness. NGS, including the Roche/454 Genome Sequencer FLX Instrument, the ABI SOLiD System and the Illumina Genome Analyser, was a powerful tool and was untilized in many researching areas, including re-sequencing, micro-RNA expression profiling, DNA methylation, especially *de novo* transcriptome sequencing for non-model organisms [5-13]. Recently, based on the NGS technology, the transcriptome of many species such as chestnut [9], coral larval [5], sweetpotato [11], ginseng root [10], chickpea [12] and saccharina japonica [13] had been analyzed, which has accelerated our understanding of the complexity of expression, regulation and networks of gene in model and non-model organisms.

Ramie (*Boehmeria nivea*), popularly named as “Chinagrass”, is a perennial diploid (2n = 28) herbaceous plant belonging to the family of *Urticaceae* and is an important natural fiber crop. Ramie fibers extracted from stem bast possess characteristics such as smooth texture, long strands and excellent tensile strength, which makes ramie to be widely planted in China, India, and other Southeast Asian and Pacific Rim countries. In China, ramie is the second major fiber crop and its growth acreage and fiber production are surpassed only by those of cotton. Ramie has a vigorous vegetative growth and can be harvested three times per year in China, and up to six times per year in well-watered cultivation environments in Philippines, which allow ramie to produce a high yield of vegetative fiber. Therefore, understanding the processes regulating vegetative growth and development of ramie is valuable. Moreover, the ramie bast fiber, involved in sugar metabolism and cellulose synthesis, is an important organ, which has the value of biological research for organogenesis and evolution. During the last decade, a large number of transcriptomic and genomic sequences became available in model organisms, such as *Arabidopsis* and rice, which has greatly improved the understanding of the complexity of growth and development in higher plants. However, for ramie, only 418 EST sequences have been deposited in the GenBank database (as of August 2012). Obviously, the public available data is far from sufficient to understand the molecular mechanisms involved in vegetative development and fiber biosynthesis in ramie. Therefore, extensive transcriptomic sequence data are essential for ramie, which can be used to discover a large number of new genes.

In the present study, we utilized the Illumina paired-end sequencing technology to characterize the transcriptome of ramie and identify the cellulose synthase (CesA) gene. Non-normalized cDNA collections from different types of tissues were used to generate a broad survey of genes associated with ramie vegetative growth and development. To the best of our knowledge, this study is the first exploration to characterize the transcriptome of ramie through the analysis of large-scale transcript sequences resulting from Illumina paired-end sequencing.

## Result

### Illumina paired-end sequencing and *de novo* assembly

In order to characterize the transcriptome of ramie and generate a broad survey of genes associated with ramie vegetative growth and development, total RNA was extracted from several vegetative tissues including leaf, root, stem bast, stem xylem and stem shoot at vegetative stages. Using Illumina paired-end sequencing technology, each sequencing feature can yield 2 × 90 bp independent reads from the either end of a DNA fragment. In this study, a total of 57,471,036 raw sequencing reads with the length of 90 bp were generated from a 200 bp insert library.

**Figure 1:**
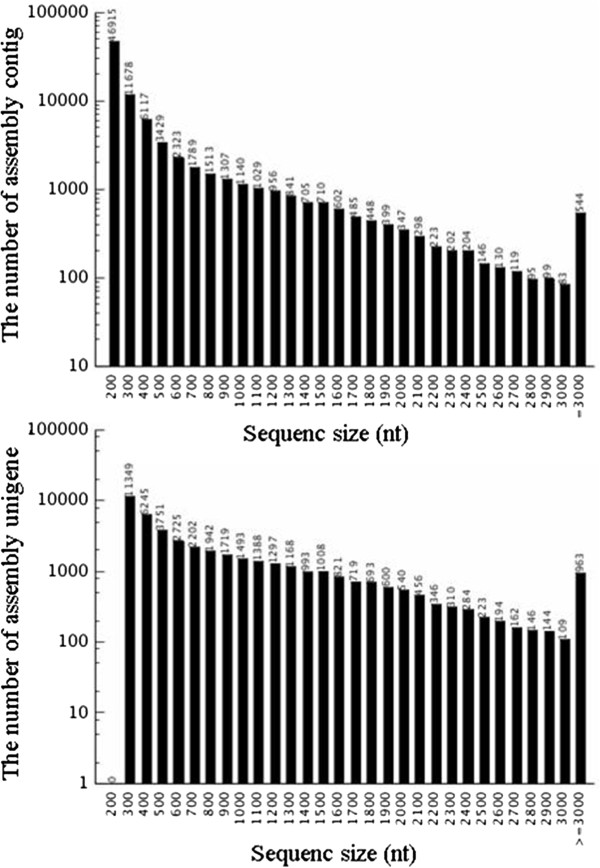
Length distribution of assembled contigs and unigenes.

An assembler, Trinity developed specifically for use with next-generation short-read sequences [14], was employed for *de novo* assembly. After stringent quality checking and data cleaning, approximately 53 million high quality reads were obtained with 97.46% Q20 bases (base quality more than 20). Based on the high quality reads, 84,876 contigs were assembled with an average length of 393 bp ranging from 100 to 17,496 bp. Of these, 44.8% and 11.6% contigs had a length more than 200 bp and 1000 bp, respectively (Figure 1).

**Figure 2:**
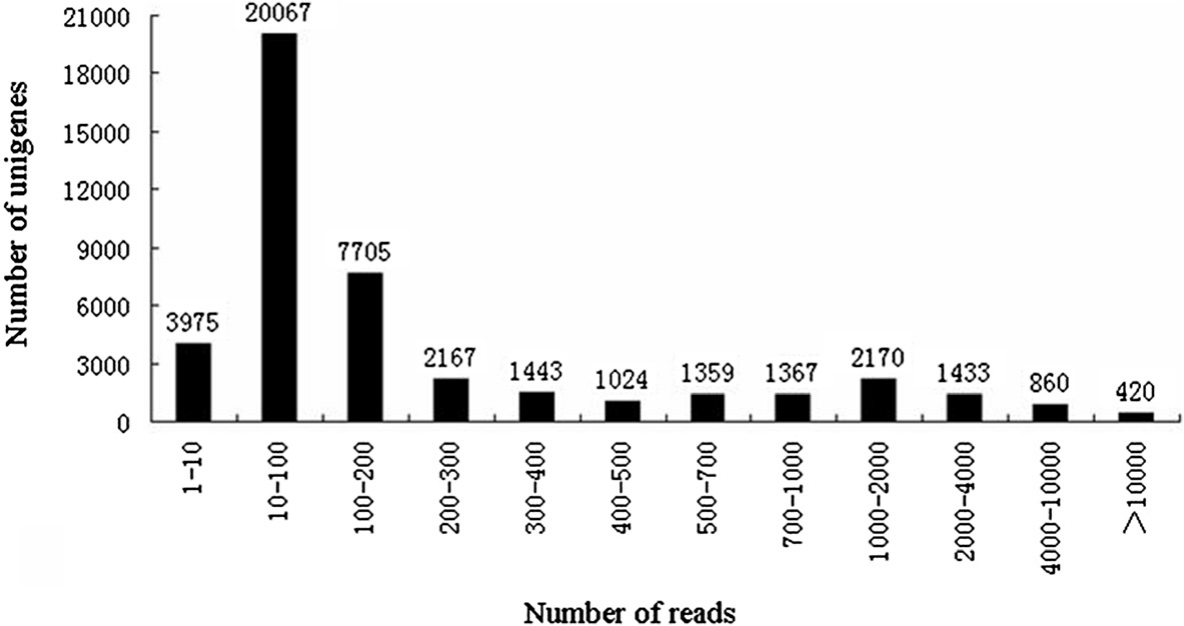
**Assessment of assembly quality.** Distribution of unique mapped reads of the assembled unigenes.

To obtain the unigenes, the paired-end reads were realigned to contigs and these contigs in one transcript were assembled by the Trinity and gained the sequence not being extended on the either end were defined as unigenes. Then the TGICL [15] was used to get rid of redundant unigene and to further assemble all the unigenes to form a single set of non-redundant unigenes. Finally the *de novo* assembly yielded 43,990 unigenes with an average length of 824 bp and a total length of 36.26 Mb. The length of assembled unigenes ranged from 200 to 17,496 bp. There were 11,349 unigenes (25.80%) with length no more than 300 bp, 9,966 unigenes (22.66%) with length varying from 301 to 500 bp, 10,081 unigenes (22.92%) in the length range of 501 to 1000 bp, and 12,564 unigenes (28.57%) with length more than 1000 bp (Figure 1). To evaluate the quality of the assembled unigenes, all the usable sequencing reads were realigned to the unigenes. The sequencing depth ranged from 0.1 to 132,686 folds, with an average of 62.98 folds. About 91.0% of the unigenes were realigned by more than 10 reads and 45.4% were remapped by more than 100 reads (Figure 2).

Sequence orientations of all unigenes were pridicted via ESTScan or BLAST (Basic Local Alignment Search Tool) with an E-value threshold of 10^-5^ in the NCBI database of non-redundant protein (Nr), along with the Swiss-Prot protein database, the Kyoto Encyclopedia of Genes and Genomes (KEGG) database and the Clusters of Orthologous Groups (COG) database. Finally, sequence orientation of 34251 (77.8%) unigenes was predicted and 9739 (22.2%) unigenes sequence orientation is still unknown.

### Functional annotation by searching against public databases

**Figure 3:**
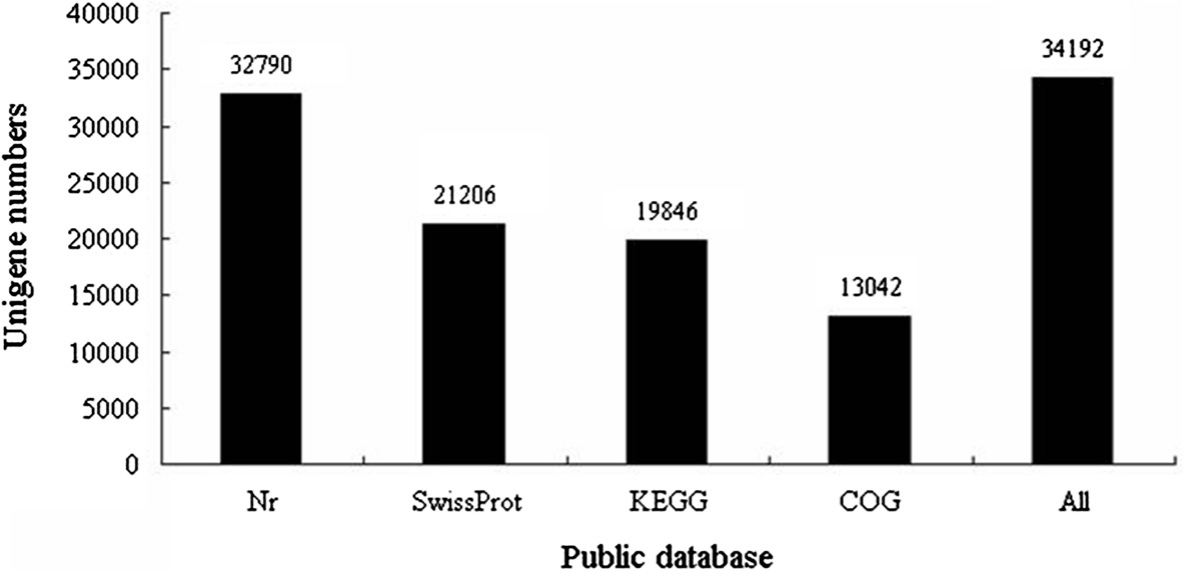
The unigene number annotated in four public database searched.

**Figure 4:**
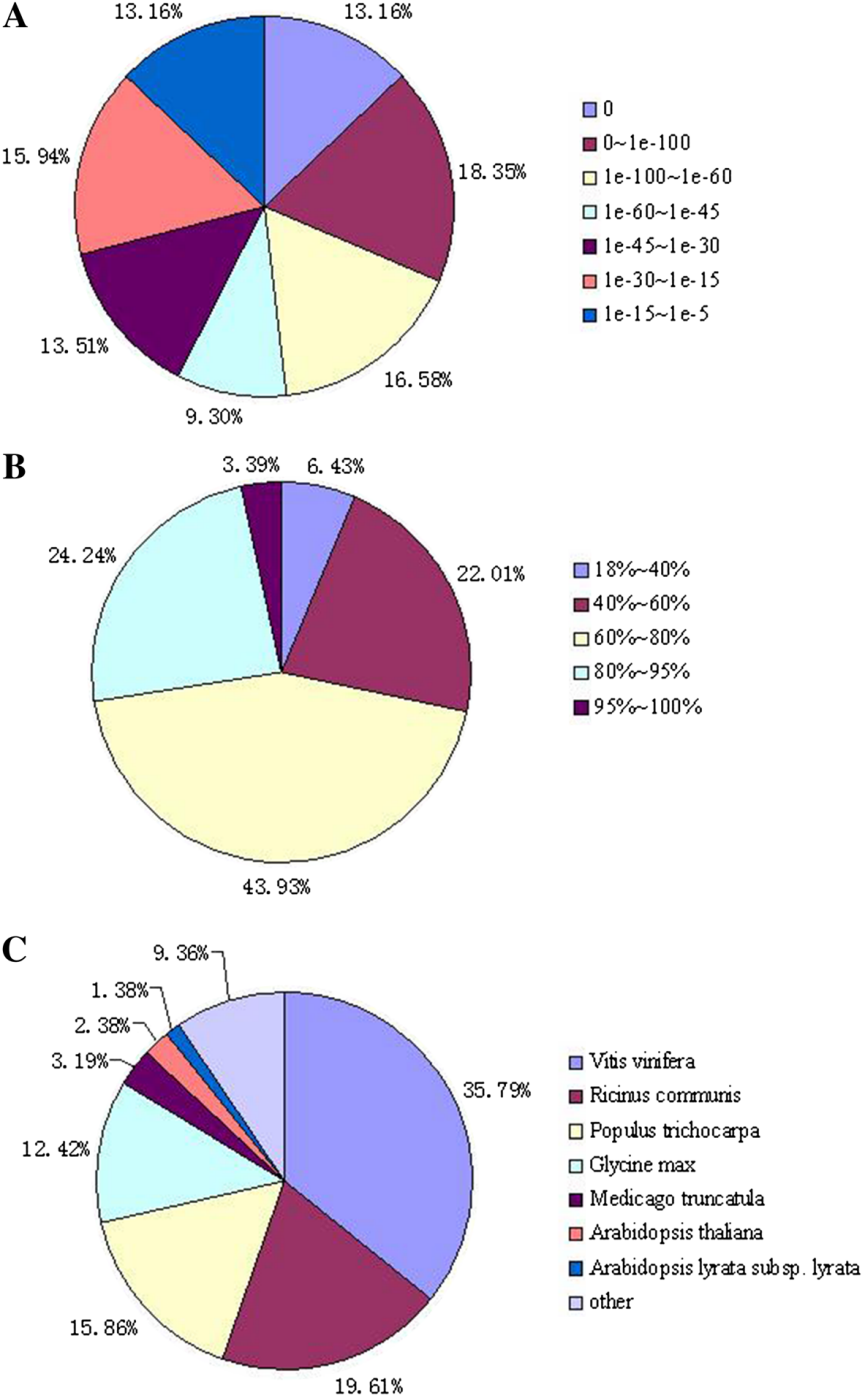
**Characteristics of similarity search of unigenes against Nr databases.** (**A**) E-value distribution of BLAST hits for each unigene with a cutoff E-value of 1.0E-5. (**B**) Similarity distribution of the top BLAST hits for each unigene. (**C**) Species distribution of the top BLAST hits for each unigenes in Nr dababase.

For validation and annotation of the assembled unigenes, sequence similarity search was conducted in the Nr database, the COG database, the Swiss-Prot protein database, and the KEGG database [16,17] with an E-value threshold of 10^-5^. The results indicated that out of 43,990 unigenes, 32,790 (74.5%), 21,206 (48.2%), 19,846 (45.1%) and 13,042 (29.6%) unigenes showed significant similarity to known proteins in Nr, SwissProt, KEGG and COG databases, respectively (Figure 3). Together, 34,192 (77.7%) unigenes showed similarity to known proteins in four databases mentioned above. The E-value distribution of the top hits in the Nr database revealed that 57.39% of the mapped sequences showed significant homology (less than 1.0E-45) (Figure 4A), and 71.56% and 27.63% of the sequences with similarities greater than 60% and 80%, respectively, were found (Figure 4B). Interestingly, 35.79% of the unigenes showed significant homology with sequence of *Vitis vinifera* and 19.61%, 15.86% and 12.42% of the mapped sequences have a significant similarity with the sequence of *Ricinus communis*, *Populus trichocarpa* and *Glycine max*, respectively (Figure 4C).

### Functional classification by GO and COG

**Figure 5:**
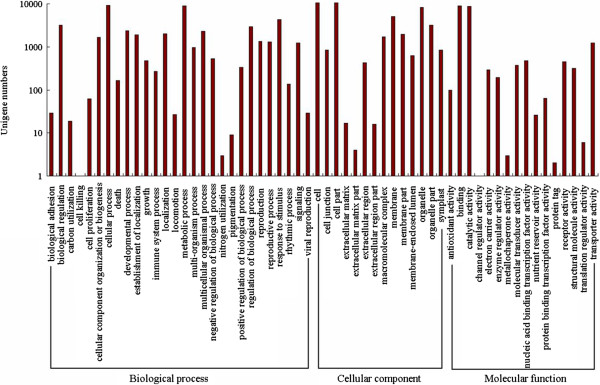
**Gene Ontology classifications of assembled unigenes.** The results are summarized in three main categories: Biological process, Cellular component and Molecular function.

Gene Ontology (GO) is an international standardized gene functional classification system which offers a dynamic-updated controlled vocabulary and a strictly defined concept to comprehensively describe the properties of genes and their products in any organism. GO has three ontologies: Molecular function, Cellular component and Biological process. On the basis of Nr annotation, the Blast2GO program [18] was used to obtain GO annotation for the unigenes annotated by the Nr database. Then the WEGO software [19] was used to perform GO functional classification for these unigenes. In total, 16,050 unigenes (36.5%) with BLAST matches to known proteins were assigned to GO classes with 111,333 functional terms. Of these, assignments to the biological process made up the majority (45,848, 44.18%), followed by molecular function (44,282, 39.77%) and cellular component (21,203, 19.05%, Figure 5).

**Figure 6:**
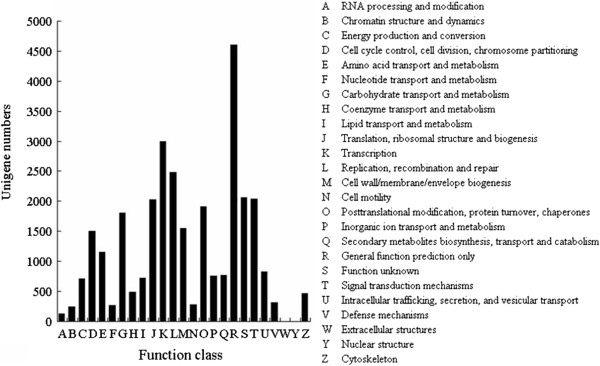
Histogram presentation of clusters of orthologous groups (COG) classification.

The Clusters of Orthologous Groups (COG) is a database where the orthologous gene products were classified. Every protein in the COG database is assumed to be evolved from an ancestor protein, and the whole database is built on coding proteins with complete genome as well as system evolution relationships of bacteria, algae and eukaryotes. All unigenes were aligned to the COG database to predict and classify potential functions. Totally, 13,042 genes (29.6%) were assigned to the 25 COG classifications (Figure 6). Some unigenes were assigned to several COG categories, which lead to a total of 30140 sequences assigned in 25 COG categories. Among the 25 COG categories, the cluster of General function prediction (4,606, 15.28%) represented the largest group, followed by Transcription (2,999, 9.95%), Replication, recombination and repair (2,481, 8.23%), Function unknown(2,061, 6.84%), Signal transduction mechanisms (2,038, 6.76%), Translation, ribosomal structure and biogenesis (2,023, 6.71%), Posttranslational modification, protein turnover, chaperones (1,913, 6.35%), Carbohydrate transport and metabolism (1,810, 6.01%) and Cell cycle control, cell division, chromosome partitioning (1,507, 5.00%), Amino acid transport and metabolism (1,158, 3.84%), whereas only a few unigenes were assigned to Nulcear structure and Extracellular structure (Figure 6).

### Metabolic pathway analysis by KEGG

The Kyoto Encyclopedia of Genes and Genomes (KEGG) Pathway database records the networks of molecular interactions in the cells, and variants of them specific to particular organisms. Pathway-based analysis helps us to further understand the biological functions and interactions of genes. First, based on a comparison against the KEGG database using BLASTx with an E-value threshold of 10^-5^, 19,846 (45.1%) sequences of the 43,990 unigenes were found to have significant matches in the database and were assigned to 126 KEGG pathways. For some genes being assigned to several KEGG pathways, there were 21,603 sequence hit in 126 KEGG pathways together. Of the 126 KEGG pathways, there were 25 pathways with over 200 unigenes assigned. The pathway of RNA transport was assigned the most unigene (1,287, 5.96%), followed by plant-pathogen interaction (1,147, 5.31%), endocytosis (979, 4.53%), glycerophospholipid metabolism (947, 4.38%), mRNA surveillance pathway (942, 4.36%), plant hormone signal transduction (928, 4.30%), ether lipid metabolism (799, 3.70%), spliceosome(745, 3.45%), starch and sucrose metabolism(565, 2.62%), protein processing in endoplasmic reticulum (493, 2.28%), whereas only no more than 10 unigenes were assigned to C5-branched dibasic acid metabolism, biotin metabolism, caffeine metabolism and betalain biosynthesis (Additional file 1: Table S1).

### Identification of the genes encoding cellulose synthase (CesA genes)

As an important natural fiber, ramie fiber development is a major research area with a focus on improving the fiber yield and quality. Cellulose is a major component of the ramie fiber, and its content in the fiber significantly influences the yield and quality of ramie fiber. Out of 43,990 genes *de novo* assembled, 34,192 unigenes were annotated their function based on the sequence similarity search against the public databases. By searching the annotation with the keyword “cellulose synthase” from these 34,192 genes, 51 CesA genes were identified. Among these 51 genes, 30 unigenes have a significant homology (less than 1.0E-50) with the CesA gene of other species; 22 genes encoding proteins have their orthologous protein with more than 80% sequence similarity; 21 ramie CesA genes are homologous with the CesA gene of *Vitis vinifera* (Additional file 2: Table S2). Additionally, the conserved domain of ramie 51 CesA genes encoding protein was searched from the conserved domain database (CCD) and the result showed that 23 CesA gene-encoded proteins possessed the conserved domain of cellulose synthase. Interestingly, there are 12 genes assigned into the KEGG pathway of starch and sucrose metabolism involved in cellulose biosynthesis, and 39 genes were not assigned to any pathway (Additional file 2: Table S2).

**Figure 7:**
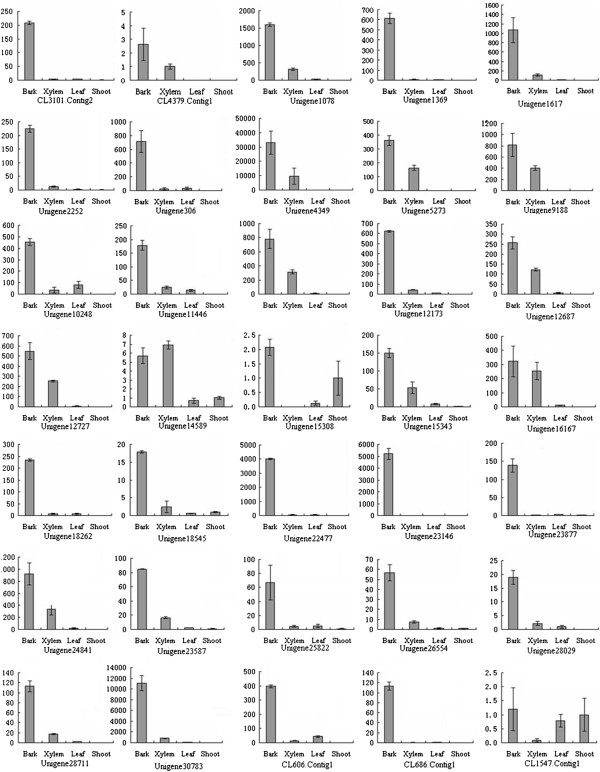
**The expression pattern of CesA genes expressed with relative high level in stem bark.** The B, X, L and S of X axis represent the stem bark, stem xylem, shoot and leaf, respectively.

In order to identify the potential candidates of the CesA genes involved in bast fiber biosynthesis, the expression levels of all 51 CesA genes in the stem bark, stem xylem, stem shoot and leaves were analyzed by RT-qPCR assay. The result showed that there were 36 CesA genes with relatively high expression levels in the stem bark from which the fiber was extracted (Additional file 2: Table S2). Among the 36 genes expressed in the bark, 33 genes showed higher expression levels in the bark than in other tissues (Figure 7); the Unigene14589 displayed a relatively high expression level in the stem bark and xylem and CL1547.contig1 showed similarly high expression levels in the stem bark, shoot and leaves, respectively (Figure 7); while the Unigene 21178 showed a constitutive expression in stem bark, shoot, xylem and leaf (Additional file 2: Table S2).

## Discussion

### Characterization of the ramie transcriptome and the discovery of 43,990 new genes

With the development of sequencing technology, many plants such as *Arabidopsis*, rice, maize and sorghum have had their complete genome sequence [20-23], which has accelerated the research on functional genomics and accumulated the understanding of mechanisms that underlie plant growth and development. However, for some non-model plants and minor crops, it is infeasible to execute whole genome sequencing because of the expensive cost. The Next Generation Sequencing (NGS) provides an opportunity to mine the genes and deepen our understanding of growth and development in non-model plants. Recently, transcriptomes of scores of non-model species have been characterized [9-13], which expanded our understanding of gene expression, regulation and networks of important traits of the corresponding plants. Previously, only 418 ramie ESTs had been identified in database despite it being an important natural fiber crop. The lack on ramie gene severely hindered our understanding of ramie growth and fiber development, which presented an obstacle to improve the fiber yield and quality. In our study, the ramie transcriptome was characterized and in all 43,990 genes were newly discovered in ramie. Sequence orientation of approximately 78% of the unigenes discovered in this study was ascertained, along with the annotation of their function by searching against public databases. In addition, the functions of the unigenes were classified by COG and GO and the metabolic pathways were ascertained by using the KEGG database. Because of this study, the number of genes identified in ramie achieved a major leap from 418 to 43,990. These results will immensely help us to explore the major genes for important agronomic traits in ramie, and further understand their regulation mechanisms, especially bast-fiber formation and development mechanism.

In our study, the transcriptome sequencing was completed by using the Illumina Genome Analyser system platform HiSeq2000. HiSeq2000 can provide the sequence with a read-length of 90 bp, which is longer than that provided by other sequencing platform, such as GAII. In addition, the raw reads were stringently filtered before the *de novo* assembly. Theoretically, the reads with adaptor contamination can still be used after trimming the adaptor sequence. However, considering that the distance of two reads of paired-end was utilized to assemble the sequence by the software, there is an inaccurate distance between two paired-end reads with a length smaller than 90 bp, which may lead to a wrong assembly. In order to attain the sequences with high quality, all reads with adaptor contamination were discarded. Therefore, our transcriptome sequence has better quality than that of other species reported by previous studies [9-13], which was evident in several aspects. First, the average length of the unigenes was 824 bp which was far longer than that of other transcriptome sequences. Second, approximately 78% of the genes discovered in this study were successfully annotated for their functions. The ratio of genes annotated function was higher than that found in previous studies. Furthermore, we arrived at a higher ratio of genes assigned to the corresponding KEGG pathways. It was obvious that the transcriptome sequence generated in this study will be valuable for further ramie research.

Sequence similarities presented new clues for determining the phylogenetic relationship among ramie, *Vitis vinifera*, *Glycine max*, *Ricinus communis* and *Populus trichocarpa.*

Significant sequence similarities of 35.79%, 19.61%, 15.86% and 12.42% between ramie and *Vitis vinifera*, *Ricinus communis*, *Populus trichocarpa* and *Glycine max*, respectively, were observed in present study. *Ricinus communi*s and *Populus trichocarpa* belong to Malpighiales. *Glycine max* belongs to the order of Fabales. Ramie is classified into Urticaceae of Rosales. In plant taxonomy, Malpighiales, Fabales, Rosales are commonly placed in the superorder of rosids [24-26]. The close relationship among Malpighiales, Fabales and Rosales was further confirmed based on the evidence of sequence similarity evidence among the corresponding species of the three orders. However, the order of Vitaceae, including the species *Vitis vinifera*, had an unclear taxonomic position in the plant phylogenetic system. In the obsolete Angiosperm Phylogeny Group I (APG I) system, the Vitaceae was not classified [24]. In the revised yet no longer used APG II system, the Vitaceae was also unplaced to order, but only included in the superorder of rosids [25]. In the revised and updated APG III system, the rosids was classified as three subgroups: Vitales, fabids and malvids [26]. Malpighiales, Fabales and Rosales commonly belong to the fabids subgroup and Vitaceae belongs to Vitales. In other words, the relationship among Malpighiales, Fabales and Rosales is considered closer than that between Vitales and Rosales. However, in this study, new evidence at the molecular level has provided a contradictory taxonomic result. The ratio of transcriptome sequence similarity between ramie and *Vitis vinifera* was one-fold higher than that between ramie and the other three species, which suggests that the relationship between Vitales and Rosales is likely closer than that between Rosales, Malpighiales and Fabales.

### Potential candidate CesA genes involved in bast fiber biosynthesis

The cellulose is a chain of glucose residues, and is the principal component of the plant cell walls. Biosynthesis of cellulose can be dissected as three steps: initiation of the sugar chain, elongation, and termination of the sugar chain [27]. The initiation and elongation cellulose is performed by cellulose synthase which is a rosette-shaped enzyme complex in the plant cell plasma membrane [28]. The catalytic subunits of cellulose synthase (CesAs) are central catalysts involved in the generation of plant cell wall cellulose [29,30]. CesA genes have been extensively studied in plants such as *Arabidopsis*, rice, cotton and barley [31-34]. Ramie bast fiber has highly enriched cellulose. However, only one ramie CesA gene has been identified based on the RACE technology [35]. In this study, with the help of transcriptome analysis and gene functional annotation, a total of 51 CesA genes were identified. Twenty-three CesA gene-encoded proteins have conserved domain of cellulose synthase. However, beacuse many EST sequences assembled in this study were not the full-length cDNA and protein sequences predicted according to these ESTs were incomplete, there are 28 CesA gene-encoded proteins that were not found their conserved domain. On the basis of the KEGG database searching, 12 CesA genes were assigned to the pathway of cellulose biosynthesis. Moreover, analysis of the expression pattern of 51 CesA genes showed that there are 36 genes with high expression levels in the stem bark. Of these 36 genes, 33 genes showed higher expression levels in the bark than in other tissues. It is likely that these 36 CesA genes are responsible for the biosynthesis of ramie fiber. Therefore, the identification of these genes will be helpful in further understanding of the mechanisms of ramie fiber development.

## Conclusion

In this study, the ramie transcriptome was first characterized by *de novo* sequencing without the presence of a reference genome using Illumina paired-end sequencing technology. In all 43,990 genes with excellent sequence qualities were identified, which achieved a great leap in the knowledge of expressed sequence information of ramie. The substantial amount of transcripts obtained will certainly accelerate the understanding of the ramie growth and development mechanism, along with providing a strong basis for future genomic research. Furthermore, 51 CesA genes were mined in our research and their expression patterns were analyzed. Thirty-six CesA genes displayed relatively high expression levels in the stem bark, 33 of which showed higher expression in the bark than in other tissues. Identification of the CesA gene expressed in the stem bark will present an opportunity to understand the formation and development of ramie bast fiber, which provide a foundation for ramie breeding to improve the fiber yield and quality.

## Methods

### Plant material and RNA extraction

The elite ramie cultivar “Zhongzhu 1” was grown in the experimental field of the Institute of Bast Fiber Crops, Chinese Academy of Agricultural Sciences, Changsha, China in 2012. Samples of tissues including leaves, root, stem bast, stem xylem and stem shoot were collected from 10-day-old seedling, 30-day-old ramie which have a vigorous vegetative growth and 60-day-old ramie whose fiber is about to ripeness, respectively. The sampled tissues were immediately frozen in liquid nitrogen and stored at −80° until use. Total RNAs were extracted from each tissue of ramie of three growth stages using TRIzol reagent (Transgene Company, Illkirch Graffenstaden Cedex, France) according to the manufacturer’s protocol. A total of 20 μg of RNA was equally pooled from the five tissues for cDNA library preparation. The residual RNA was used for RT-qPCR.

### cDNA library construction and sequencing

Illumina sequencing was performed at Beijing Genomics Institute (BGI)-Shenzhen, Shenzhen, China (http://www.genomics.cn/index.php) by using the HiSeq™ 2000 platform according to the manufacturer’s instructions (Illumina, San Diego, CA). Briefly, poly (A) RNA was isolated from 20 μg of total RNA using Sera-mag Magnetic Oligo (dT) Beads (Illumina). To avoid priming bias when synthesizing cDNA, the purified mRNA was first fragmented into small pieces. Then the double-stranded cDNA was synthesized using the SuperScript Double-Stranded cDNA Synthesis kit (Invitrogen, Camarillo, CA) with random hexamer (N6) primers (Illumina). The synthesized cDNA was subjected to end-repair and phosphorylation using T4 DNA polymerase, Klenow DNA polymerase and T4 PNK. These repaired cDNA fragments were 3’ adenylated using Klenow Exo- (3’ to 5’ exo minus, Illumina). Illumina Paired-end adapters were ligated to the ends of these 3’-adenylated cDNA fragments. To select a size range of templates for downstream enrichment, the products of ligation reaction were purified on a 2% TAE-agarose gel (Certified Low-Range Ultra Agarose, Biorad). A range of cDNA fragments (200 ± 25 bp) was excised from the gel. Fifteen rounds of PCR amplification were performed to enrich the purified cDNA template using PCR Primer PE 1.0 and PE 2.0 (Illumina) with Phusion DNA Polymerase. The cDNA library was constructed with a fragment length range of 200 bp (±25 bp). Finally, after validating on an Agilent Technologies 2100 Bioanalyzer using the Agilent DNA 1000 chip kit, the cDNA library was sequenced on a PE flow cell using Illumina Genome Analyzer HiSeq 2000, and the workflow was as follows: template hybridization, isothermal amplification, linearization, blocking, sequencing primer hybridization, and sequencing on the sequencer for Read 1. After completion of the first read, the templates can be regenerated in situ to enable a second 90 bp read from the opposite end of the fragments, i.e., the newly sequenced strands are stripped off and the complementary strands are bridge amplified to form clusters. Once the original templates are cleaved and removed, the reverse strands undergo sequencing-by-synthesis. The sequencing data are deposited in NCBI Sequence Read Archive (SRA, http://www.ncbi.nlm.nih.gov/Traces/sra) with accession number SRA057664.

### Data filtering and *de novo* assembly

The quality requirement for *de novo* transcriptome sequencing is far higher than that for re-sequencing, because sequencing errors can create difficulties for the short-read assembly algorithm. We therefore carried out a stringent filtering process. Firstly, we removed reads that do not pass the built-in Illumina’s software Failed-Chastity filter according to the relation “failed-chastity ≤ 1”, using a chastity threshold of 0.6, on the first 25cycles. Secondly, we discarded all reads with adaptor contamination. Thirdly, we ruled out low-quality reads with more than 5% ambiguous sequences “N”. Finally, the reads with more than 20% Q < 20 bases were also removed. *De novo* assembly was carried out using Trinity [14]. Additionally, if there are mutli-duplication’s reads, only one read copy will be retained for assembly and redundant duplication reads be eliminated. Trinity assembled some reads which had overlapped nucleic acid sequence and generated contigs. To obtain the unigene, the paired-end reads were realigned to contigs, which can identify different contigs in the same transcript and ascertain the interval among these contigs. Then, these contigs in one transcript were assembled by the Trinity and gained the sequence not being extended on either end defined as unigenes. Then the TGICL [15] is used to get rid of redundant unigene and further assemble all the unigenes to form a single set of non-redundant unigenes.

Finally, BLASTx alignment (E value < 10^-5^) between unigenes and protein databases like NCBI non-redundant protein (Nr) database http://www.ncbi.nlm.nih.gov, Swiss-Prot protein database http://www.expasy.ch/sprot, the Kyoto Encyclopedia of Genes and Genomes (KEGG) pathway database http://www.genome.jp/kegg, and the Cluster of Orthologous Groups database http://www.ncbi.nlm.nih.gov/COG was performed, and the best aligning results were used to decide the sequence direction of unigenes. If the results of different databases conflicted with each other, a priority order of Nr, Swiss-Prot, KEGG and COG should be followed when deciding the sequence direction of unigenes. When a unigene happened to be unaligned to none of the above databases, ESTScan software [36] was used to predict its coding regions as well as to decide its sequence direction.

### Gene annotation and analysis

For annotation of unigenes using various bioinformatics approaches, the unigenes were firstly searched against the Nr, COG, KEGG and Swiss-Prot protein database using local BLASTx with E value cutoff of 10^-5^. To estimate the number of annotated unigenes that matched to unique genes in the two databases, these files were filtered. With Nr annotation, Blast2GO program [18] was used to get GO annotation according to molecular function, biological process and cellular component ontologies http://www.geneontology.org. The sequences of the unigenes were also aligned to the COG database to predict and classify possible functions. Pathway assignments were carried out according to the Kyoto Encyclopedia of Genes and Genomes pathway database [37] also using BLASTx with E value threshold of 10^-5^. The conserved domain of CesA gene encoding protein was searched from the conserved domain database (CCD) in NCBI [38].

### RT-qPCR analysis of CesA genes expression pattern

The RNA extracted for stem bark, xylem, shoot and leaf was used for RT-qPCR. For each sample, first-strand cDNAs were reverse-transcribed from RNAs treated with DNase I (Fermentas, Canada) using M-MuLV Reverse Transcriptase (Fermentas, Canada) according to the manufacturer’s instructions. RT-qPCR was performed using an optical 96-well plate with an iQ5 multicolor real time PCR system (Bio-RAD, USA). Each reaction contained 1 μL of cDNA template, 10 nM gene-specific primers, 10 μL of SYBR Premix Ex Taq, and 0.4 μL of ROX Reference Dye (FINNZYMES, Finland) in a final volume of 20 μL. The 18S rRNA gene was selected for the endogenous control [35]. The primer sequence of CesA genes and 18S rRNA gene were listed in Additional file 3: Table S3. The thermal cycle used was as follows: 95°C for 15 min, followed by 40 cycles of 95°C for 10 s, 55°C for 20s and 72°C for 30 s. RT-qPCR was performed in triplicate for each sample. Relative expression levels were determined as described previously [39].

## Abbreviations

GO: Gene ontology; RT-qPCR: Quantitative real-time polymerase chain reaction; CesA: cellulose synthase; KEGG: Kyoto Encyclopedia of Genes and Genomes Pathway database; EST: expressed sequence tag; NGS: next generation sequencing; Nr database: NCBI non-redundant protein database; COG: Cluster of Orthologous Groups; CCD: conserved domain database; BLAST: Basic Local Alignment Search Tool.

## Competing interests

The authors have declared that no competing interests exist.

## Authors’ contributions

LT and TS conceived and designed the experiment. LT and ZS performed the experiment. TQ, CP and YY helped to prepare the reagents and materials. LT carried out the data analysis and wrote the manuscript. All authors read and approved the final manuscript.

## Supplementary Material

Additional file 1: Table S1 Pathway assignment based on KEGG. Click here for file

Additional file 2: Table S2 CesA gene identified in ramie. Click here for file

Additional file 3: Table S3 Primer sequence of CesA and 18S RNA genes used for RT-qPCR. Click here for file
