# Supplementary material for: De novo assembly and characterization of transcriptome using Illumina paired-end sequencing and identification of CesA gene in ramie (Boehmeria nivea L. Gaud)
Source: BMC Genomics. 2013 Feb 26;14:125. doi: 10.1186/1471-2164-14-125 (PMC3610122; doi:10.1186/1471-2164-14-125)
Supplement: Additional file 1: Table S1 — Pathway assignment based on KEGG. [file 1471-2164-14-125-S1.doc]

|  | **Pathway** | **Count (19846)** | **Pathway ID** |
| --- | --- | --- | --- |
| 1 | [RNA transport](../../../../C:%5CDocuments%20and%20Settings%5CAdministrator%5C%E6%A1%8C%E9%9D%A2%5C%E6%96%B0%E5%BB%BA%20Microsoft%20Excel%20%E5%B7%A5%E4%BD%9C%E8%A1%A8.xls#Sheet1!gene3%23RANGE!gene3) | 1287 | ko03013 |
| 2 | [Plant-pathogen interaction](../../../../C:%5CDocuments%20and%20Settings%5CAdministrator%5C%E6%A1%8C%E9%9D%A2%5C%E6%96%B0%E5%BB%BA%20Microsoft%20Excel%20%E5%B7%A5%E4%BD%9C%E8%A1%A8.xls#Sheet1!gene4%23RANGE!gene4) | 1147 | ko04626 |
| 3 | [Endocytosis](../../../../C:%5CDocuments%20and%20Settings%5CAdministrator%5C%E6%A1%8C%E9%9D%A2%5C%E6%96%B0%E5%BB%BA%20Microsoft%20Excel%20%E5%B7%A5%E4%BD%9C%E8%A1%A8.xls#Sheet1!gene5%23RANGE!gene5) | 979 | ko04144 |
| 4 | [Glycerophospholipid metabolism](../../../../C:%5CDocuments%20and%20Settings%5CAdministrator%5C%E6%A1%8C%E9%9D%A2%5C%E6%96%B0%E5%BB%BA%20Microsoft%20Excel%20%E5%B7%A5%E4%BD%9C%E8%A1%A8.xls#Sheet1!gene6%23RANGE!gene6) | 947 | ko00564 |
| 5 | [mRNA surveillance pathway](../../../../C:%5CDocuments%20and%20Settings%5CAdministrator%5C%E6%A1%8C%E9%9D%A2%5C%E6%96%B0%E5%BB%BA%20Microsoft%20Excel%20%E5%B7%A5%E4%BD%9C%E8%A1%A8.xls#Sheet1!gene7%23RANGE!gene7) | 942 | ko03015 |
| 6 | [Plant hormone signal transduction](../../../../C:%5CDocuments%20and%20Settings%5CAdministrator%5C%E6%A1%8C%E9%9D%A2%5C%E6%96%B0%E5%BB%BA%20Microsoft%20Excel%20%E5%B7%A5%E4%BD%9C%E8%A1%A8.xls#Sheet1!gene8%23RANGE!gene8) | 928 | ko04075 |
| 7 | [Ether lipid metabolism](../../../../C:%5CDocuments%20and%20Settings%5CAdministrator%5C%E6%A1%8C%E9%9D%A2%5C%E6%96%B0%E5%BB%BA%20Microsoft%20Excel%20%E5%B7%A5%E4%BD%9C%E8%A1%A8.xls#Sheet1!gene9%23RANGE!gene9) | 799 | ko00565 |
| 8 | [Spliceosome](../../../../C:%5CDocuments%20and%20Settings%5CAdministrator%5C%E6%A1%8C%E9%9D%A2%5C%E6%96%B0%E5%BB%BA%20Microsoft%20Excel%20%E5%B7%A5%E4%BD%9C%E8%A1%A8.xls#Sheet1!gene10%23RANGE!gene10) | 745 | ko03040 |
| 9 | [Starch and sucrose metabolism](../../../../C:%5CDocuments%20and%20Settings%5CAdministrator%5C%E6%A1%8C%E9%9D%A2%5C%E6%96%B0%E5%BB%BA%20Microsoft%20Excel%20%E5%B7%A5%E4%BD%9C%E8%A1%A8.xls#Sheet1!gene11%23RANGE!gene11) | 565 | ko00500 |
| 10 | [Protein processing in endoplasmic reticulum](../../../../C:%5CDocuments%20and%20Settings%5CAdministrator%5C%E6%A1%8C%E9%9D%A2%5C%E6%96%B0%E5%BB%BA%20Microsoft%20Excel%20%E5%B7%A5%E4%BD%9C%E8%A1%A8.xls#Sheet1!gene12%23RANGE!gene12) | 493 | ko04141 |
| 11 | [Purine metabolism](../../../../C:%5CDocuments%20and%20Settings%5CAdministrator%5C%E6%A1%8C%E9%9D%A2%5C%E6%96%B0%E5%BB%BA%20Microsoft%20Excel%20%E5%B7%A5%E4%BD%9C%E8%A1%A8.xls#Sheet1!gene13%23RANGE!gene13) | 426 | ko00230 |
| 12 | [Pyrimidine metabolism](../../../../C:%5CDocuments%20and%20Settings%5CAdministrator%5C%E6%A1%8C%E9%9D%A2%5C%E6%96%B0%E5%BB%BA%20Microsoft%20Excel%20%E5%B7%A5%E4%BD%9C%E8%A1%A8.xls#Sheet1!gene14%23RANGE!gene14) | 387 | ko00240 |
| 13 | [Ribosome biogenesis in eukaryotes](../../../../C:%5CDocuments%20and%20Settings%5CAdministrator%5C%E6%A1%8C%E9%9D%A2%5C%E6%96%B0%E5%BB%BA%20Microsoft%20Excel%20%E5%B7%A5%E4%BD%9C%E8%A1%A8.xls#Sheet1!gene15%23RANGE!gene15) | 362 | ko03008 |
| 14 | [Ribosome](../../../../C:%5CDocuments%20and%20Settings%5CAdministrator%5C%E6%A1%8C%E9%9D%A2%5C%E6%96%B0%E5%BB%BA%20Microsoft%20Excel%20%E5%B7%A5%E4%BD%9C%E8%A1%A8.xls#Sheet1!gene16%23RANGE!gene16) | 354 | ko03010 |
| 15 | [Ubiquitin mediated proteolysis](../../../../C:%5CDocuments%20and%20Settings%5CAdministrator%5C%E6%A1%8C%E9%9D%A2%5C%E6%96%B0%E5%BB%BA%20Microsoft%20Excel%20%E5%B7%A5%E4%BD%9C%E8%A1%A8.xls#Sheet1!gene17%23RANGE!gene17) | 348 | ko04120 |
| 16 | [Phenylpropanoid biosynthesis](../../../../C:%5CDocuments%20and%20Settings%5CAdministrator%5C%E6%A1%8C%E9%9D%A2%5C%E6%96%B0%E5%BB%BA%20Microsoft%20Excel%20%E5%B7%A5%E4%BD%9C%E8%A1%A8.xls#Sheet1!gene18%23RANGE!gene18) | 343 | ko00940 |
| 17 | [RNA degradation](../../../../C:%5CDocuments%20and%20Settings%5CAdministrator%5C%E6%A1%8C%E9%9D%A2%5C%E6%96%B0%E5%BB%BA%20Microsoft%20Excel%20%E5%B7%A5%E4%BD%9C%E8%A1%A8.xls#Sheet1!gene19%23RANGE!gene19) | 341 | ko03018 |
| 18 | [Pentose and glucuronate interconversions](../../../../C:%5CDocuments%20and%20Settings%5CAdministrator%5C%E6%A1%8C%E9%9D%A2%5C%E6%96%B0%E5%BB%BA%20Microsoft%20Excel%20%E5%B7%A5%E4%BD%9C%E8%A1%A8.xls#Sheet1!gene20%23RANGE!gene20) | 320 | ko00040 |
| 19 | [Oxidative phosphorylation](../../../../C:%5CDocuments%20and%20Settings%5CAdministrator%5C%E6%A1%8C%E9%9D%A2%5C%E6%96%B0%E5%BB%BA%20Microsoft%20Excel%20%E5%B7%A5%E4%BD%9C%E8%A1%A8.xls#Sheet1!gene21%23RANGE!gene21) | 256 | ko00190 |
| 20 | [Phagosome](../../../../C:%5CDocuments%20and%20Settings%5CAdministrator%5C%E6%A1%8C%E9%9D%A2%5C%E6%96%B0%E5%BB%BA%20Microsoft%20Excel%20%E5%B7%A5%E4%BD%9C%E8%A1%A8.xls#Sheet1!gene22%23RANGE!gene22) | 238 | ko04145 |
| 21 | [RNA polymerase](../../../../C:%5CDocuments%20and%20Settings%5CAdministrator%5C%E6%A1%8C%E9%9D%A2%5C%E6%96%B0%E5%BB%BA%20Microsoft%20Excel%20%E5%B7%A5%E4%BD%9C%E8%A1%A8.xls#Sheet1!gene23%23RANGE!gene23) | 233 | ko03020 |
| 22 | [Glycolysis / Gluconeogenesis](../../../../C:%5CDocuments%20and%20Settings%5CAdministrator%5C%E6%A1%8C%E9%9D%A2%5C%E6%96%B0%E5%BB%BA%20Microsoft%20Excel%20%E5%B7%A5%E4%BD%9C%E8%A1%A8.xls#Sheet1!gene24%23RANGE!gene24) | 225 | ko00010 |
| 23 | [Amino sugar and nucleotide sugar metabolism](../../../../C:%5CDocuments%20and%20Settings%5CAdministrator%5C%E6%A1%8C%E9%9D%A2%5C%E6%96%B0%E5%BB%BA%20Microsoft%20Excel%20%E5%B7%A5%E4%BD%9C%E8%A1%A8.xls#Sheet1!gene25%23RANGE!gene25) | 219 | ko00520 |
| 24 | [Flavonoid biosynthesis](../../../../C:%5CDocuments%20and%20Settings%5CAdministrator%5C%E6%A1%8C%E9%9D%A2%5C%E6%96%B0%E5%BB%BA%20Microsoft%20Excel%20%E5%B7%A5%E4%BD%9C%E8%A1%A8.xls#Sheet1!gene26%23RANGE!gene26) | 212 | ko00941 |
| 25 | [ABC transporters](../../../../C:%5CDocuments%20and%20Settings%5CAdministrator%5C%E6%A1%8C%E9%9D%A2%5C%E6%96%B0%E5%BB%BA%20Microsoft%20Excel%20%E5%B7%A5%E4%BD%9C%E8%A1%A8.xls#Sheet1!gene27%23RANGE!gene27) | 211 | ko02010 |
| 26 | [Cysteine and methionine metabolism](../../../../C:%5CDocuments%20and%20Settings%5CAdministrator%5C%E6%A1%8C%E9%9D%A2%5C%E6%96%B0%E5%BB%BA%20Microsoft%20Excel%20%E5%B7%A5%E4%BD%9C%E8%A1%A8.xls#Sheet1!gene28%23RANGE!gene28) | 181 | ko00270 |
| 27 | [Stilbenoid, diarylheptanoid and gingerol biosynthesis](../../../../C:%5CDocuments%20and%20Settings%5CAdministrator%5C%E6%A1%8C%E9%9D%A2%5C%E6%96%B0%E5%BB%BA%20Microsoft%20Excel%20%E5%B7%A5%E4%BD%9C%E8%A1%A8.xls#Sheet1!gene29%23RANGE!gene29) | 180 | ko00945 |
| 28 | [Nucleotide excision repair](../../../../C:%5CDocuments%20and%20Settings%5CAdministrator%5C%E6%A1%8C%E9%9D%A2%5C%E6%96%B0%E5%BB%BA%20Microsoft%20Excel%20%E5%B7%A5%E4%BD%9C%E8%A1%A8.xls#Sheet1!gene30%23RANGE!gene30) | 179 | ko03420 |
| 29 | [Peroxisome](../../../../C:%5CDocuments%20and%20Settings%5CAdministrator%5C%E6%A1%8C%E9%9D%A2%5C%E6%96%B0%E5%BB%BA%20Microsoft%20Excel%20%E5%B7%A5%E4%BD%9C%E8%A1%A8.xls#Sheet1!gene31%23RANGE!gene31) | 174 | ko04146 |
| 30 | [Zeatin biosynthesis](../../../../C:%5CDocuments%20and%20Settings%5CAdministrator%5C%E6%A1%8C%E9%9D%A2%5C%E6%96%B0%E5%BB%BA%20Microsoft%20Excel%20%E5%B7%A5%E4%BD%9C%E8%A1%A8.xls#Sheet1!gene32%23RANGE!gene32) | 173 | ko00908 |
| 31 | [Carotenoid biosynthesis](../../../../C:%5CDocuments%20and%20Settings%5CAdministrator%5C%E6%A1%8C%E9%9D%A2%5C%E6%96%B0%E5%BB%BA%20Microsoft%20Excel%20%E5%B7%A5%E4%BD%9C%E8%A1%A8.xls#Sheet1!gene33%23RANGE!gene33) | 165 | ko00906 |
| 32 | [Pyruvate metabolism](../../../../C:%5CDocuments%20and%20Settings%5CAdministrator%5C%E6%A1%8C%E9%9D%A2%5C%E6%96%B0%E5%BB%BA%20Microsoft%20Excel%20%E5%B7%A5%E4%BD%9C%E8%A1%A8.xls#Sheet1!gene34%23RANGE!gene34) | 160 | ko00620 |
| 33 | [Circadian rhythm - plant](../../../../C:%5CDocuments%20and%20Settings%5CAdministrator%5C%E6%A1%8C%E9%9D%A2%5C%E6%96%B0%E5%BB%BA%20Microsoft%20Excel%20%E5%B7%A5%E4%BD%9C%E8%A1%A8.xls#Sheet1!gene35%23RANGE!gene35) | 157 | ko04712 |
| 34 | [Basal transcription factors](../../../../C:%5CDocuments%20and%20Settings%5CAdministrator%5C%E6%A1%8C%E9%9D%A2%5C%E6%96%B0%E5%BB%BA%20Microsoft%20Excel%20%E5%B7%A5%E4%BD%9C%E8%A1%A8.xls#Sheet1!gene36%23RANGE!gene36) | 151 | ko03022 |
| 35 | [Phenylalanine metabolism](../../../../C:%5CDocuments%20and%20Settings%5CAdministrator%5C%E6%A1%8C%E9%9D%A2%5C%E6%96%B0%E5%BB%BA%20Microsoft%20Excel%20%E5%B7%A5%E4%BD%9C%E8%A1%A8.xls#Sheet1!gene37%23RANGE!gene37) | 150 | ko00360 |
| 36 | [Photosynthesis](../../../../C:%5CDocuments%20and%20Settings%5CAdministrator%5C%E6%A1%8C%E9%9D%A2%5C%E6%96%B0%E5%BB%BA%20Microsoft%20Excel%20%E5%B7%A5%E4%BD%9C%E8%A1%A8.xls#Sheet1!gene38%23RANGE!gene38) | 146 | ko00195 |
| 37 | [Phosphatidylinositol signaling system](../../../../C:%5CDocuments%20and%20Settings%5CAdministrator%5C%E6%A1%8C%E9%9D%A2%5C%E6%96%B0%E5%BB%BA%20Microsoft%20Excel%20%E5%B7%A5%E4%BD%9C%E8%A1%A8.xls#Sheet1!gene39%23RANGE!gene39) | 144 | ko04070 |
| 38 | [Homologous recombination](../../../../C:%5CDocuments%20and%20Settings%5CAdministrator%5C%E6%A1%8C%E9%9D%A2%5C%E6%96%B0%E5%BB%BA%20Microsoft%20Excel%20%E5%B7%A5%E4%BD%9C%E8%A1%A8.xls#Sheet1!gene40%23RANGE!gene40) | 144 | ko03440 |
| 39 | [Inositol phosphate metabolism](../../../../C:%5CDocuments%20and%20Settings%5CAdministrator%5C%E6%A1%8C%E9%9D%A2%5C%E6%96%B0%E5%BB%BA%20Microsoft%20Excel%20%E5%B7%A5%E4%BD%9C%E8%A1%A8.xls#Sheet1!gene41%23RANGE!gene41) | 144 | ko00562 |
| 40 | [Carbon fixation in photosynthetic organisms](../../../../C:%5CDocuments%20and%20Settings%5CAdministrator%5C%E6%A1%8C%E9%9D%A2%5C%E6%96%B0%E5%BB%BA%20Microsoft%20Excel%20%E5%B7%A5%E4%BD%9C%E8%A1%A8.xls#Sheet1!gene42%23RANGE!gene42) | 144 | ko00710 |
| 41 | [Arginine and proline metabolism](../../../../C:%5CDocuments%20and%20Settings%5CAdministrator%5C%E6%A1%8C%E9%9D%A2%5C%E6%96%B0%E5%BB%BA%20Microsoft%20Excel%20%E5%B7%A5%E4%BD%9C%E8%A1%A8.xls#Sheet1!gene43%23RANGE!gene43) | 142 | ko00330 |
| 42 | [Limonene and pinene degradation](../../../../C:%5CDocuments%20and%20Settings%5CAdministrator%5C%E6%A1%8C%E9%9D%A2%5C%E6%96%B0%E5%BB%BA%20Microsoft%20Excel%20%E5%B7%A5%E4%BD%9C%E8%A1%A8.xls#Sheet1!gene44%23RANGE!gene44) | 139 | ko00903 |
| 43 | [Galactose metabolism](../../../../C:%5CDocuments%20and%20Settings%5CAdministrator%5C%E6%A1%8C%E9%9D%A2%5C%E6%96%B0%E5%BB%BA%20Microsoft%20Excel%20%E5%B7%A5%E4%BD%9C%E8%A1%A8.xls#Sheet1!gene45%23RANGE!gene45) | 139 | ko00052 |
| 44 | [Terpenoid backbone biosynthesis](../../../../C:%5CDocuments%20and%20Settings%5CAdministrator%5C%E6%A1%8C%E9%9D%A2%5C%E6%96%B0%E5%BB%BA%20Microsoft%20Excel%20%E5%B7%A5%E4%BD%9C%E8%A1%A8.xls#Sheet1!gene46%23RANGE!gene46) | 139 | ko00900 |
| 45 | [Tyrosine metabolism](../../../../C:%5CDocuments%20and%20Settings%5CAdministrator%5C%E6%A1%8C%E9%9D%A2%5C%E6%96%B0%E5%BB%BA%20Microsoft%20Excel%20%E5%B7%A5%E4%BD%9C%E8%A1%A8.xls#Sheet1!gene47%23RANGE!gene47) | 136 | ko00350 |
| 46 | [Fructose and mannose metabolism](../../../../C:%5CDocuments%20and%20Settings%5CAdministrator%5C%E6%A1%8C%E9%9D%A2%5C%E6%96%B0%E5%BB%BA%20Microsoft%20Excel%20%E5%B7%A5%E4%BD%9C%E8%A1%A8.xls#Sheet1!gene48%23RANGE!gene48) | 136 | ko00051 |
| 47 | [Regulation of autophagy](../../../../C:%5CDocuments%20and%20Settings%5CAdministrator%5C%E6%A1%8C%E9%9D%A2%5C%E6%96%B0%E5%BB%BA%20Microsoft%20Excel%20%E5%B7%A5%E4%BD%9C%E8%A1%A8.xls#Sheet1!gene49%23RANGE!gene49) | 134 | ko04140 |
| 48 | [Glutathione metabolism](../../../../C:%5CDocuments%20and%20Settings%5CAdministrator%5C%E6%A1%8C%E9%9D%A2%5C%E6%96%B0%E5%BB%BA%20Microsoft%20Excel%20%E5%B7%A5%E4%BD%9C%E8%A1%A8.xls#Sheet1!gene50%23RANGE!gene50) | 130 | ko00480 |
| 49 | [Cyanoamino acid metabolism](../../../../C:%5CDocuments%20and%20Settings%5CAdministrator%5C%E6%A1%8C%E9%9D%A2%5C%E6%96%B0%E5%BB%BA%20Microsoft%20Excel%20%E5%B7%A5%E4%BD%9C%E8%A1%A8.xls#Sheet1!gene51%23RANGE!gene51) | 128 | ko00460 |
| 50 | [Glycine, serine and threonine metabolism](../../../../C:%5CDocuments%20and%20Settings%5CAdministrator%5C%E6%A1%8C%E9%9D%A2%5C%E6%96%B0%E5%BB%BA%20Microsoft%20Excel%20%E5%B7%A5%E4%BD%9C%E8%A1%A8.xls#Sheet1!gene52%23RANGE!gene52) | 127 | ko00260 |
| 51 | [Other glycan degradation](../../../../C:%5CDocuments%20and%20Settings%5CAdministrator%5C%E6%A1%8C%E9%9D%A2%5C%E6%96%B0%E5%BB%BA%20Microsoft%20Excel%20%E5%B7%A5%E4%BD%9C%E8%A1%A8.xls#Sheet1!gene53%23RANGE!gene53) | 123 | ko00511 |
| 52 | [Pentose phosphate pathway](../../../../C:%5CDocuments%20and%20Settings%5CAdministrator%5C%E6%A1%8C%E9%9D%A2%5C%E6%96%B0%E5%BB%BA%20Microsoft%20Excel%20%E5%B7%A5%E4%BD%9C%E8%A1%A8.xls#Sheet1!gene54%23RANGE!gene54) | 117 | ko00030 |
| 53 | [DNA replication](../../../../C:%5CDocuments%20and%20Settings%5CAdministrator%5C%E6%A1%8C%E9%9D%A2%5C%E6%96%B0%E5%BB%BA%20Microsoft%20Excel%20%E5%B7%A5%E4%BD%9C%E8%A1%A8.xls#Sheet1!gene55%23RANGE!gene55) | 116 | ko03030 |
| 54 | [Valine, leucine and isoleucine degradation](../../../../C:%5CDocuments%20and%20Settings%5CAdministrator%5C%E6%A1%8C%E9%9D%A2%5C%E6%96%B0%E5%BB%BA%20Microsoft%20Excel%20%E5%B7%A5%E4%BD%9C%E8%A1%A8.xls#Sheet1!gene56%23RANGE!gene56) | 113 | ko00280 |
| 55 | [Base excision repair](../../../../C:%5CDocuments%20and%20Settings%5CAdministrator%5C%E6%A1%8C%E9%9D%A2%5C%E6%96%B0%E5%BB%BA%20Microsoft%20Excel%20%E5%B7%A5%E4%BD%9C%E8%A1%A8.xls#Sheet1!gene57%23RANGE!gene57) | 113 | ko03410 |
| 56 | [Porphyrin and chlorophyll metabolism](../../../../C:%5CDocuments%20and%20Settings%5CAdministrator%5C%E6%A1%8C%E9%9D%A2%5C%E6%96%B0%E5%BB%BA%20Microsoft%20Excel%20%E5%B7%A5%E4%BD%9C%E8%A1%A8.xls#Sheet1!gene58%23RANGE!gene58) | 112 | ko00860 |
| 57 | [alpha-Linolenic acid metabolism](../../../../C:%5CDocuments%20and%20Settings%5CAdministrator%5C%E6%A1%8C%E9%9D%A2%5C%E6%96%B0%E5%BB%BA%20Microsoft%20Excel%20%E5%B7%A5%E4%BD%9C%E8%A1%A8.xls#Sheet1!gene59%23RANGE!gene59) | 110 | ko00592 |
| 58 | [Flavone and flavonol biosynthesis](../../../../C:%5CDocuments%20and%20Settings%5CAdministrator%5C%E6%A1%8C%E9%9D%A2%5C%E6%96%B0%E5%BB%BA%20Microsoft%20Excel%20%E5%B7%A5%E4%BD%9C%E8%A1%A8.xls#Sheet1!gene60%23RANGE!gene60) | 108 | ko00944 |
| 59 | [Aminoacyl-tRNA biosynthesis](../../../../C:%5CDocuments%20and%20Settings%5CAdministrator%5C%E6%A1%8C%E9%9D%A2%5C%E6%96%B0%E5%BB%BA%20Microsoft%20Excel%20%E5%B7%A5%E4%BD%9C%E8%A1%A8.xls#Sheet1!gene61%23RANGE!gene61) | 108 | ko00970 |
| 60 | [Mismatch repair](../../../../C:%5CDocuments%20and%20Settings%5CAdministrator%5C%E6%A1%8C%E9%9D%A2%5C%E6%96%B0%E5%BB%BA%20Microsoft%20Excel%20%E5%B7%A5%E4%BD%9C%E8%A1%A8.xls#Sheet1!gene62%23RANGE!gene62) | 103 | ko03430 |
| 61 | [Ascorbate and aldarate metabolism](../../../../C:%5CDocuments%20and%20Settings%5CAdministrator%5C%E6%A1%8C%E9%9D%A2%5C%E6%96%B0%E5%BB%BA%20Microsoft%20Excel%20%E5%B7%A5%E4%BD%9C%E8%A1%A8.xls#Sheet1!gene63%23RANGE!gene63) | 102 | ko00053 |
| 62 | [Glycerolipid metabolism](../../../../C:%5CDocuments%20and%20Settings%5CAdministrator%5C%E6%A1%8C%E9%9D%A2%5C%E6%96%B0%E5%BB%BA%20Microsoft%20Excel%20%E5%B7%A5%E4%BD%9C%E8%A1%A8.xls#Sheet1!gene64%23RANGE!gene64) | 102 | ko00561 |
| 63 | [Glycosylphosphatidylinositol(GPI)-anchor biosynthesis](../../../../C:%5CDocuments%20and%20Settings%5CAdministrator%5C%E6%A1%8C%E9%9D%A2%5C%E6%96%B0%E5%BB%BA%20Microsoft%20Excel%20%E5%B7%A5%E4%BD%9C%E8%A1%A8.xls#Sheet1!gene65%23RANGE!gene65) | 101 | ko00563 |
| 64 | [Ubiquinone and other terpenoid-quinone biosynthesis](../../../../C:%5CDocuments%20and%20Settings%5CAdministrator%5C%E6%A1%8C%E9%9D%A2%5C%E6%96%B0%E5%BB%BA%20Microsoft%20Excel%20%E5%B7%A5%E4%BD%9C%E8%A1%A8.xls#Sheet1!gene66%23RANGE!gene66) | 98 | ko00130 |
| 65 | [Propanoate metabolism](../../../../C:%5CDocuments%20and%20Settings%5CAdministrator%5C%E6%A1%8C%E9%9D%A2%5C%E6%96%B0%E5%BB%BA%20Microsoft%20Excel%20%E5%B7%A5%E4%BD%9C%E8%A1%A8.xls#Sheet1!gene67%23RANGE!gene67) | 91 | ko00640 |
| 66 | [Protein export](../../../../C:%5CDocuments%20and%20Settings%5CAdministrator%5C%E6%A1%8C%E9%9D%A2%5C%E6%96%B0%E5%BB%BA%20Microsoft%20Excel%20%E5%B7%A5%E4%BD%9C%E8%A1%A8.xls#Sheet1!gene68%23RANGE!gene68) | 89 | ko03060 |
| 67 | [Glyoxylate and dicarboxylate metabolism](../../../../C:%5CDocuments%20and%20Settings%5CAdministrator%5C%E6%A1%8C%E9%9D%A2%5C%E6%96%B0%E5%BB%BA%20Microsoft%20Excel%20%E5%B7%A5%E4%BD%9C%E8%A1%A8.xls#Sheet1!gene69%23RANGE!gene69) | 89 | ko00630 |
| 68 | [beta-Alanine metabolism](../../../../C:%5CDocuments%20and%20Settings%5CAdministrator%5C%E6%A1%8C%E9%9D%A2%5C%E6%96%B0%E5%BB%BA%20Microsoft%20Excel%20%E5%B7%A5%E4%BD%9C%E8%A1%A8.xls#Sheet1!gene70%23RANGE!gene70) | 87 | ko00410 |
| 69 | [Alanine, aspartate and glutamate metabolism](../../../../C:%5CDocuments%20and%20Settings%5CAdministrator%5C%E6%A1%8C%E9%9D%A2%5C%E6%96%B0%E5%BB%BA%20Microsoft%20Excel%20%E5%B7%A5%E4%BD%9C%E8%A1%A8.xls#Sheet1!gene71%23RANGE!gene71) | 87 | ko00250 |
| 70 | [Phenylalanine, tyrosine and tryptophan biosynthesis](../../../../C:%5CDocuments%20and%20Settings%5CAdministrator%5C%E6%A1%8C%E9%9D%A2%5C%E6%96%B0%E5%BB%BA%20Microsoft%20Excel%20%E5%B7%A5%E4%BD%9C%E8%A1%A8.xls#Sheet1!gene72%23RANGE!gene72) | 83 | ko00400 |
| 71 | [Citrate cycle (TCA cycle)](../../../../C:%5CDocuments%20and%20Settings%5CAdministrator%5C%E6%A1%8C%E9%9D%A2%5C%E6%96%B0%E5%BB%BA%20Microsoft%20Excel%20%E5%B7%A5%E4%BD%9C%E8%A1%A8.xls#Sheet1!gene73%23RANGE!gene73) | 80 | ko00020 |
| 72 | [Diterpenoid biosynthesis](../../../../C:%5CDocuments%20and%20Settings%5CAdministrator%5C%E6%A1%8C%E9%9D%A2%5C%E6%96%B0%E5%BB%BA%20Microsoft%20Excel%20%E5%B7%A5%E4%BD%9C%E8%A1%A8.xls#Sheet1!gene74%23RANGE!gene74) | 79 | ko00904 |
| 73 | [SNARE interactions in vesicular transport](../../../../C:%5CDocuments%20and%20Settings%5CAdministrator%5C%E6%A1%8C%E9%9D%A2%5C%E6%96%B0%E5%BB%BA%20Microsoft%20Excel%20%E5%B7%A5%E4%BD%9C%E8%A1%A8.xls#Sheet1!gene75%23RANGE!gene75) | 79 | ko04130 |
| 74 | [Nitrogen metabolism](../../../../C:%5CDocuments%20and%20Settings%5CAdministrator%5C%E6%A1%8C%E9%9D%A2%5C%E6%96%B0%E5%BB%BA%20Microsoft%20Excel%20%E5%B7%A5%E4%BD%9C%E8%A1%A8.xls#Sheet1!gene76%23RANGE!gene76) | 78 | ko00910 |
| 75 | [Isoquinoline alkaloid biosynthesis](../../../../C:%5CDocuments%20and%20Settings%5CAdministrator%5C%E6%A1%8C%E9%9D%A2%5C%E6%96%B0%E5%BB%BA%20Microsoft%20Excel%20%E5%B7%A5%E4%BD%9C%E8%A1%A8.xls#Sheet1!gene77%23RANGE!gene77) | 77 | ko00950 |
| 76 | [Cutin, suberine and wax biosynthesis](../../../../C:%5CDocuments%20and%20Settings%5CAdministrator%5C%E6%A1%8C%E9%9D%A2%5C%E6%96%B0%E5%BB%BA%20Microsoft%20Excel%20%E5%B7%A5%E4%BD%9C%E8%A1%A8.xls#Sheet1!gene78%23RANGE!gene78) | 76 | ko00073 |
| 77 | [N-Glycan biosynthesis](../../../../C:%5CDocuments%20and%20Settings%5CAdministrator%5C%E6%A1%8C%E9%9D%A2%5C%E6%96%B0%E5%BB%BA%20Microsoft%20Excel%20%E5%B7%A5%E4%BD%9C%E8%A1%A8.xls#Sheet1!gene79%23RANGE!gene79) | 74 | ko00510 |
| 78 | [Sphingolipid metabolism](../../../../C:%5CDocuments%20and%20Settings%5CAdministrator%5C%E6%A1%8C%E9%9D%A2%5C%E6%96%B0%E5%BB%BA%20Microsoft%20Excel%20%E5%B7%A5%E4%BD%9C%E8%A1%A8.xls#Sheet1!gene80%23RANGE!gene80) | 73 | ko00600 |
| 79 | [Pantothenate and CoA biosynthesis](../../../../C:%5CDocuments%20and%20Settings%5CAdministrator%5C%E6%A1%8C%E9%9D%A2%5C%E6%96%B0%E5%BB%BA%20Microsoft%20Excel%20%E5%B7%A5%E4%BD%9C%E8%A1%A8.xls#Sheet1!gene81%23RANGE!gene81) | 72 | ko00770 |
| 80 | [Fatty acid metabolism](../../../../C:%5CDocuments%20and%20Settings%5CAdministrator%5C%E6%A1%8C%E9%9D%A2%5C%E6%96%B0%E5%BB%BA%20Microsoft%20Excel%20%E5%B7%A5%E4%BD%9C%E8%A1%A8.xls#Sheet1!gene82%23RANGE!gene82) | 71 | ko00071 |
| 81 | [Lysine biosynthesis](../../../../C:%5CDocuments%20and%20Settings%5CAdministrator%5C%E6%A1%8C%E9%9D%A2%5C%E6%96%B0%E5%BB%BA%20Microsoft%20Excel%20%E5%B7%A5%E4%BD%9C%E8%A1%A8.xls#Sheet1!gene83%23RANGE!gene83) | 68 | ko00300 |
| 82 | [Steroid biosynthesis](../../../../C:%5CDocuments%20and%20Settings%5CAdministrator%5C%E6%A1%8C%E9%9D%A2%5C%E6%96%B0%E5%BB%BA%20Microsoft%20Excel%20%E5%B7%A5%E4%BD%9C%E8%A1%A8.xls#Sheet1!gene84%23RANGE!gene84) | 68 | ko00100 |
| 83 | [Linoleic acid metabolism](../../../../C:%5CDocuments%20and%20Settings%5CAdministrator%5C%E6%A1%8C%E9%9D%A2%5C%E6%96%B0%E5%BB%BA%20Microsoft%20Excel%20%E5%B7%A5%E4%BD%9C%E8%A1%A8.xls#Sheet1!gene85%23RANGE!gene85) | 66 | ko00591 |
| 84 | [Glycosaminoglycan degradation](../../../../C:%5CDocuments%20and%20Settings%5CAdministrator%5C%E6%A1%8C%E9%9D%A2%5C%E6%96%B0%E5%BB%BA%20Microsoft%20Excel%20%E5%B7%A5%E4%BD%9C%E8%A1%A8.xls#Sheet1!gene86%23RANGE!gene86) | 62 | ko00531 |
| 85 | [Histidine metabolism](../../../../C:%5CDocuments%20and%20Settings%5CAdministrator%5C%E6%A1%8C%E9%9D%A2%5C%E6%96%B0%E5%BB%BA%20Microsoft%20Excel%20%E5%B7%A5%E4%BD%9C%E8%A1%A8.xls#Sheet1!gene87%23RANGE!gene87) | 62 | ko00340 |
| 86 | [Natural killer cell mediated cytotoxicity](../../../../C:%5CDocuments%20and%20Settings%5CAdministrator%5C%E6%A1%8C%E9%9D%A2%5C%E6%96%B0%E5%BB%BA%20Microsoft%20Excel%20%E5%B7%A5%E4%BD%9C%E8%A1%A8.xls#Sheet1!gene88%23RANGE!gene88) | 60 | ko04650 |
| 87 | [Benzoxazinoid biosynthesis](../../../../C:%5CDocuments%20and%20Settings%5CAdministrator%5C%E6%A1%8C%E9%9D%A2%5C%E6%96%B0%E5%BB%BA%20Microsoft%20Excel%20%E5%B7%A5%E4%BD%9C%E8%A1%A8.xls#Sheet1!gene89%23RANGE!gene89) | 60 | ko00402 |
| 88 | [Biosynthesis of unsaturated fatty acids](../../../../C:%5CDocuments%20and%20Settings%5CAdministrator%5C%E6%A1%8C%E9%9D%A2%5C%E6%96%B0%E5%BB%BA%20Microsoft%20Excel%20%E5%B7%A5%E4%BD%9C%E8%A1%A8.xls#Sheet1!gene90%23RANGE!gene90) | 57 | ko01040 |
| 89 | [Tryptophan metabolism](../../../../C:%5CDocuments%20and%20Settings%5CAdministrator%5C%E6%A1%8C%E9%9D%A2%5C%E6%96%B0%E5%BB%BA%20Microsoft%20Excel%20%E5%B7%A5%E4%BD%9C%E8%A1%A8.xls#Sheet1!gene91%23RANGE!gene91) | 56 | ko00380 |
| 90 | [Proteasome](../../../../C:%5CDocuments%20and%20Settings%5CAdministrator%5C%E6%A1%8C%E9%9D%A2%5C%E6%96%B0%E5%BB%BA%20Microsoft%20Excel%20%E5%B7%A5%E4%BD%9C%E8%A1%A8.xls#Sheet1!gene92%23RANGE!gene92) | 56 | ko03050 |
| 91 | [Valine, leucine and isoleucine biosynthesis](../../../../C:%5CDocuments%20and%20Settings%5CAdministrator%5C%E6%A1%8C%E9%9D%A2%5C%E6%96%B0%E5%BB%BA%20Microsoft%20Excel%20%E5%B7%A5%E4%BD%9C%E8%A1%A8.xls#Sheet1!gene93%23RANGE!gene93) | 56 | ko00290 |
| 92 | [Isoflavonoid biosynthesis](../../../../C:%5CDocuments%20and%20Settings%5CAdministrator%5C%E6%A1%8C%E9%9D%A2%5C%E6%96%B0%E5%BB%BA%20Microsoft%20Excel%20%E5%B7%A5%E4%BD%9C%E8%A1%A8.xls#Sheet1!gene94%23RANGE!gene94) | 55 | ko00943 |
| 93 | [Sesquiterpenoid and triterpenoid biosynthesis](../../../../C:%5CDocuments%20and%20Settings%5CAdministrator%5C%E6%A1%8C%E9%9D%A2%5C%E6%96%B0%E5%BB%BA%20Microsoft%20Excel%20%E5%B7%A5%E4%BD%9C%E8%A1%A8.xls#Sheet1!gene95%23RANGE!gene95) | 54 | ko00909 |
| 94 | [Sulfur metabolism](../../../../C:%5CDocuments%20and%20Settings%5CAdministrator%5C%E6%A1%8C%E9%9D%A2%5C%E6%96%B0%E5%BB%BA%20Microsoft%20Excel%20%E5%B7%A5%E4%BD%9C%E8%A1%A8.xls#Sheet1!gene96%23RANGE!gene96) | 53 | ko00920 |
| 95 | [Fatty acid biosynthesis](../../../../C:%5CDocuments%20and%20Settings%5CAdministrator%5C%E6%A1%8C%E9%9D%A2%5C%E6%96%B0%E5%BB%BA%20Microsoft%20Excel%20%E5%B7%A5%E4%BD%9C%E8%A1%A8.xls#Sheet1!gene97%23RANGE!gene97) | 52 | ko00061 |
| 96 | [Lysine degradation](../../../../C:%5CDocuments%20and%20Settings%5CAdministrator%5C%E6%A1%8C%E9%9D%A2%5C%E6%96%B0%E5%BB%BA%20Microsoft%20Excel%20%E5%B7%A5%E4%BD%9C%E8%A1%A8.xls#Sheet1!gene98%23RANGE!gene98) | 50 | ko00310 |
| 97 | [Butanoate metabolism](../../../../C:%5CDocuments%20and%20Settings%5CAdministrator%5C%E6%A1%8C%E9%9D%A2%5C%E6%96%B0%E5%BB%BA%20Microsoft%20Excel%20%E5%B7%A5%E4%BD%9C%E8%A1%A8.xls#Sheet1!gene99%23RANGE!gene99) | 49 | ko00650 |
| 98 | [Fatty acid elongation](../../../../C:%5CDocuments%20and%20Settings%5CAdministrator%5C%E6%A1%8C%E9%9D%A2%5C%E6%96%B0%E5%BB%BA%20Microsoft%20Excel%20%E5%B7%A5%E4%BD%9C%E8%A1%A8.xls#Sheet1!gene100%23RANGE!gene100) | 49 | ko00062 |
| 99 | [Riboflavin metabolism](../../../../C:%5CDocuments%20and%20Settings%5CAdministrator%5C%E6%A1%8C%E9%9D%A2%5C%E6%96%B0%E5%BB%BA%20Microsoft%20Excel%20%E5%B7%A5%E4%BD%9C%E8%A1%A8.xls#Sheet1!gene101%23RANGE!gene101) | 48 | ko00740 |
| 100 | [Tropane, piperidine and pyridine alkaloid biosynthesis](../../../../C:%5CDocuments%20and%20Settings%5CAdministrator%5C%E6%A1%8C%E9%9D%A2%5C%E6%96%B0%E5%BB%BA%20Microsoft%20Excel%20%E5%B7%A5%E4%BD%9C%E8%A1%A8.xls#Sheet1!gene102%23RANGE!gene102) | 48 | ko00960 |
| 101 | [Brassinosteroid biosynthesis](../../../../C:%5CDocuments%20and%20Settings%5CAdministrator%5C%E6%A1%8C%E9%9D%A2%5C%E6%96%B0%E5%BB%BA%20Microsoft%20Excel%20%E5%B7%A5%E4%BD%9C%E8%A1%A8.xls#Sheet1!gene103%23RANGE!gene103) | 44 | ko00905 |
| 102 | [Glucosinolate biosynthesis](../../../../C:%5CDocuments%20and%20Settings%5CAdministrator%5C%E6%A1%8C%E9%9D%A2%5C%E6%96%B0%E5%BB%BA%20Microsoft%20Excel%20%E5%B7%A5%E4%BD%9C%E8%A1%A8.xls#Sheet1!gene104%23RANGE!gene104) | 43 | ko00966 |
| 103 | [Glycosphingolipid biosynthesis - ganglio series](../../../../C:%5CDocuments%20and%20Settings%5CAdministrator%5C%E6%A1%8C%E9%9D%A2%5C%E6%96%B0%E5%BB%BA%20Microsoft%20Excel%20%E5%B7%A5%E4%BD%9C%E8%A1%A8.xls#Sheet1!gene105%23RANGE!gene105) | 41 | ko00604 |
| 104 | [Non-homologous end-joining](../../../../C:%5CDocuments%20and%20Settings%5CAdministrator%5C%E6%A1%8C%E9%9D%A2%5C%E6%96%B0%E5%BB%BA%20Microsoft%20Excel%20%E5%B7%A5%E4%BD%9C%E8%A1%A8.xls#Sheet1!gene106%23RANGE!gene106) | 41 | ko03450 |
| 105 | [Photosynthesis - antenna proteins](../../../../C:%5CDocuments%20and%20Settings%5CAdministrator%5C%E6%A1%8C%E9%9D%A2%5C%E6%96%B0%E5%BB%BA%20Microsoft%20Excel%20%E5%B7%A5%E4%BD%9C%E8%A1%A8.xls#Sheet1!gene107%23RANGE!gene107) | 37 | ko00196 |
| 106 | [Monoterpenoid biosynthesis](../../../../C:%5CDocuments%20and%20Settings%5CAdministrator%5C%E6%A1%8C%E9%9D%A2%5C%E6%96%B0%E5%BB%BA%20Microsoft%20Excel%20%E5%B7%A5%E4%BD%9C%E8%A1%A8.xls#Sheet1!gene108%23RANGE!gene108) | 35 | ko00902 |
| 107 | [Folate biosynthesis](../../../../C:%5CDocuments%20and%20Settings%5CAdministrator%5C%E6%A1%8C%E9%9D%A2%5C%E6%96%B0%E5%BB%BA%20Microsoft%20Excel%20%E5%B7%A5%E4%BD%9C%E8%A1%A8.xls#Sheet1!gene109%23RANGE!gene109) | 33 | ko00790 |
| 108 | [Other types of O-glycan biosynthesis](../../../../C:%5CDocuments%20and%20Settings%5CAdministrator%5C%E6%A1%8C%E9%9D%A2%5C%E6%96%B0%E5%BB%BA%20Microsoft%20Excel%20%E5%B7%A5%E4%BD%9C%E8%A1%A8.xls#Sheet1!gene110%23RANGE!gene110) | 32 | ko00514 |
| 109 | [Arachidonic acid metabolism](../../../../C:%5CDocuments%20and%20Settings%5CAdministrator%5C%E6%A1%8C%E9%9D%A2%5C%E6%96%B0%E5%BB%BA%20Microsoft%20Excel%20%E5%B7%A5%E4%BD%9C%E8%A1%A8.xls#Sheet1!gene111%23RANGE!gene111) | 30 | ko00590 |
| 110 | [One carbon pool by folate](../../../../C:%5CDocuments%20and%20Settings%5CAdministrator%5C%E6%A1%8C%E9%9D%A2%5C%E6%96%B0%E5%BB%BA%20Microsoft%20Excel%20%E5%B7%A5%E4%BD%9C%E8%A1%A8.xls#Sheet1!gene112%23RANGE!gene112) | 30 | ko00670 |
| 111 | [Vitamin B6 metabolism](../../../../C:%5CDocuments%20and%20Settings%5CAdministrator%5C%E6%A1%8C%E9%9D%A2%5C%E6%96%B0%E5%BB%BA%20Microsoft%20Excel%20%E5%B7%A5%E4%BD%9C%E8%A1%A8.xls#Sheet1!gene113%23RANGE!gene113) | 30 | ko00750 |
| 112 | [Selenocompound metabolism](../../../../C:%5CDocuments%20and%20Settings%5CAdministrator%5C%E6%A1%8C%E9%9D%A2%5C%E6%96%B0%E5%BB%BA%20Microsoft%20Excel%20%E5%B7%A5%E4%BD%9C%E8%A1%A8.xls#Sheet1!gene114%23RANGE!gene114) | 26 | ko00450 |
| 113 | [Indole alkaloid biosynthesis](../../../../C:%5CDocuments%20and%20Settings%5CAdministrator%5C%E6%A1%8C%E9%9D%A2%5C%E6%96%B0%E5%BB%BA%20Microsoft%20Excel%20%E5%B7%A5%E4%BD%9C%E8%A1%A8.xls#Sheet1!gene115%23RANGE!gene115) | 24 | ko00901 |
| 114 | [Nicotinate and nicotinamide metabolism](../../../../C:%5CDocuments%20and%20Settings%5CAdministrator%5C%E6%A1%8C%E9%9D%A2%5C%E6%96%B0%E5%BB%BA%20Microsoft%20Excel%20%E5%B7%A5%E4%BD%9C%E8%A1%A8.xls#Sheet1!gene116%23RANGE!gene116) | 23 | ko00760 |
| 115 | [Circadian rhythm - mammal](../../../../C:%5CDocuments%20and%20Settings%5CAdministrator%5C%E6%A1%8C%E9%9D%A2%5C%E6%96%B0%E5%BB%BA%20Microsoft%20Excel%20%E5%B7%A5%E4%BD%9C%E8%A1%A8.xls#Sheet1!gene117%23RANGE!gene117) | 21 | ko04710 |
| 116 | [Synthesis and degradation of ketone bodies](../../../../C:%5CDocuments%20and%20Settings%5CAdministrator%5C%E6%A1%8C%E9%9D%A2%5C%E6%96%B0%E5%BB%BA%20Microsoft%20Excel%20%E5%B7%A5%E4%BD%9C%E8%A1%A8.xls#Sheet1!gene118%23RANGE!gene118) | 18 | ko00072 |
| 117 | [Glycosphingolipid biosynthesis - globo series](../../../../C:%5CDocuments%20and%20Settings%5CAdministrator%5C%E6%A1%8C%E9%9D%A2%5C%E6%96%B0%E5%BB%BA%20Microsoft%20Excel%20%E5%B7%A5%E4%BD%9C%E8%A1%A8.xls#Sheet1!gene119%23RANGE!gene119) | 17 | ko00603 |
| 118 | [Lipoic acid metabolism](../../../../C:%5CDocuments%20and%20Settings%5CAdministrator%5C%E6%A1%8C%E9%9D%A2%5C%E6%96%B0%E5%BB%BA%20Microsoft%20Excel%20%E5%B7%A5%E4%BD%9C%E8%A1%A8.xls#Sheet1!gene120%23RANGE!gene120) | 16 | ko00785 |
| 119 | [Sulfur relay system](../../../../C:%5CDocuments%20and%20Settings%5CAdministrator%5C%E6%A1%8C%E9%9D%A2%5C%E6%96%B0%E5%BB%BA%20Microsoft%20Excel%20%E5%B7%A5%E4%BD%9C%E8%A1%A8.xls#Sheet1!gene121%23RANGE!gene121) | 15 | ko04122 |
| 120 | [Taurine and hypotaurine metabolism](../../../../C:%5CDocuments%20and%20Settings%5CAdministrator%5C%E6%A1%8C%E9%9D%A2%5C%E6%96%B0%E5%BB%BA%20Microsoft%20Excel%20%E5%B7%A5%E4%BD%9C%E8%A1%A8.xls#Sheet1!gene122%23RANGE!gene122) | 12 | ko00430 |
| 121 | [Anthocyanin biosynthesis](../../../../C:%5CDocuments%20and%20Settings%5CAdministrator%5C%E6%A1%8C%E9%9D%A2%5C%E6%96%B0%E5%BB%BA%20Microsoft%20Excel%20%E5%B7%A5%E4%BD%9C%E8%A1%A8.xls#Sheet1!gene123%23RANGE!gene123) | 11 | ko00942 |
| 122 | [Thiamine metabolism](../../../../C:%5CDocuments%20and%20Settings%5CAdministrator%5C%E6%A1%8C%E9%9D%A2%5C%E6%96%B0%E5%BB%BA%20Microsoft%20Excel%20%E5%B7%A5%E4%BD%9C%E8%A1%A8.xls#Sheet1!gene124%23RANGE!gene124) | 11 | ko00730 |
| 123 | [C5-Branched dibasic acid metabolism](../../../../C:%5CDocuments%20and%20Settings%5CAdministrator%5C%E6%A1%8C%E9%9D%A2%5C%E6%96%B0%E5%BB%BA%20Microsoft%20Excel%20%E5%B7%A5%E4%BD%9C%E8%A1%A8.xls#Sheet1!gene125%23RANGE!gene125) | 9 | ko00660 |
| 124 | [Biotin metabolism](../../../../C:%5CDocuments%20and%20Settings%5CAdministrator%5C%E6%A1%8C%E9%9D%A2%5C%E6%96%B0%E5%BB%BA%20Microsoft%20Excel%20%E5%B7%A5%E4%BD%9C%E8%A1%A8.xls#Sheet1!gene126%23RANGE!gene126) | 7 | ko00780 |
| 125 | [Caffeine metabolism](../../../../C:%5CDocuments%20and%20Settings%5CAdministrator%5C%E6%A1%8C%E9%9D%A2%5C%E6%96%B0%E5%BB%BA%20Microsoft%20Excel%20%E5%B7%A5%E4%BD%9C%E8%A1%A8.xls#Sheet1!gene127%23RANGE!gene127) | 4 | ko00232 |
| 126 | [Betalain biosynthesis](../../../../C:%5CDocuments%20and%20Settings%5CAdministrator%5C%E6%A1%8C%E9%9D%A2%5C%E6%96%B0%E5%BB%BA%20Microsoft%20Excel%20%E5%B7%A5%E4%BD%9C%E8%A1%A8.xls#Sheet1!gene128%23RANGE!gene128) | 4 | ko00965 |
