# Supplementary material for: De novo assembly and characterization of transcriptome using Illumina paired-end sequencing and identification of CesA gene in ramie (Boehmeria nivea L. Gaud)
Source: BMC Genomics. 2013 Feb 26;14:125. doi: 10.1186/1471-2164-14-125 (PMC3610122; doi:10.1186/1471-2164-14-125)
Supplement: Additional file 2: Table S2 — CesA gene identified in ramie. [file 1471-2164-14-125-S2.doc]

| Gene | Containing Conserved domain | Main tissue expressed | Orthologous gene | | | |
| --- | --- | --- | --- | --- | --- | --- |
|  |  |  | Genebank ID | Species | Identity (%) | E_value |
| CL3101.Contig2 |  | Stem bark | MTR_3g030040 | Medicago truncatula | 40.62 | 2E－64 |
| CL4379.Contig1* |  | Stem bark | LOC100243459 | Vitis vinifera | 67.24 | 0 |
| Unigene1078 |  | Stem bark | LOC100256811 | Vitis vinifera | 92.9 | 0 |
| Unigene1110 |  | Stem xylem | POPTRDRAFT_551308 | Populus trichocarpa | 86.86 | 0 |
| Unigene1369 | Glyco_tranf_GTA_type super family | Stem bark | LOC100810567 | Glycine max | 93.81 | 3E－113 |
| Unigene1617 |  | Stem bark | LOC100254408 | Vitis vinifera | 93.78 | 0 |
| Unigene2252 |  | Stem bark | LOC100819146 | Glycine max | 91.59 | 0 |
| Unigene306 |  | Stem bark | AT5G05170 | Arabidopsis thaliana | 43.36 | 6E－69 |
| Unigene4349 |  | Stem bark | POPTRDRAFT_819877 | Populus trichocarpa | 85.08 | 0 |
| Unigene5273 | Glyco_tranf_GTA_type super family | Stem bark | POPTRDRAFT_819877 | Populus trichocarpa | 52.7 | 8E－15 |
| Unigene9188 |  | Stem bark | cslA3 | Physcomitrella patens subsp. patens | 41.11 | 3E－71 |
| Unigene10248 | Glyco_tranf_GTA_type super family | Stem bark | LOC100248512 | Vitis vinifera | 31.66 | 3E－50 |
| Unigene11446* | Glyco_tranf_GTA_type super family | Stem bark | LOC100243459 | Vitis vinifera | 90.95 | 4E－132 |
| Unigene11548 | Glyco_tranf_GTA_type super family | Stem xylem | LOC100241197 | Vitis vinifera | 88.43 | 0 |
| Unigene11682 |  | Stem xylem | LOC100254408 | Vitis vinifera | 43.75 | 3E－59 |
| Unigene12047 | PLN02195 | Stem bark | LOC100261696 | Vitis vinifera | 70.11 | 6E－69 |
| Unigene12173* | PLN02248 | Stem bark | POPTRDRAFT_552489 | Populus trichocarpa | 79.73 | 7E－31 |
| Unigene12537 |  | Stem xylem | LOC100248512 | Vitis vinifera | 39.2 | 8E－94 |
| Unigene12687 |  | Stem bark | Os01g0750300 | Oryza sativa japonica | 28.75 | 8E－17 |
| Unigene12727 | Glyco_tranf_GTA_type super family | Stem bark | AT5G17420 | Arabidopsis thaliana | 30.93 | 2E－50 |
| Unigene14037 | Glyco_tranf_GTA_type super family | Stem xylem | LOC100806522 | Glycine max | 94.41 | 2E－78 |
| Unigene14589 |  | Stem xylem、bark | LOC100843829 | Brachypodium distachyon | 55.2 | 5E－37 |
| Unigene15308* | Glyco_tranf_GTA_type super family | Stem bark | POPTRDRAFT_552489 | Populus trichocarpa | 89.23 | 4E－29 |
| Unigene15343 |  | Stem bark | AT5G17420 | Arabidopsis thaliana | 85.15 | 6E－49 |
| Unigene16167 |  | Stem bark | MTR_2g035780 | Medicago truncatula | 78.46 | 7E－87 |
| Unigene16287 |  | Stem xylem | LOC100241197 | Vitis vinifera | 96.23 | 4E－25 |
| Unigene16487* |  | Shoot | LOC100243459 | Vitis vinifera | 90.4 | 2E－106 |
| Unigene16621 |  | Stem xylem | LOC100241197 | Vitis vinifera | 93.77 | 1E－154 |
| Unigene18262 | Glyco_tranf_GTA_type super family | Stem bark | LOC100806522 | Glycine max | 46.23 | 2E－38 |
| Unigene18545 | Glyco_tranf_GTA_type super family | Stem bark | LOC100806786 | Glycine max | 44.04 | 1E－35 |
| Unigene18559 | Glyco_tranf_GTA_type super family | Stem xylem | LOC100248512 | Vitis vinifera | 92.39 | 7E－107 |
| Unigene19033 |  | Stem xylem | LOC100794515 | Glycine max | 29.06 | 3E－37 |
| Unigene19910* |  | Shoot | POPTRDRAFT_552489 | Populus trichocarpa | 67.47 | 2E－19 |
| Unigene21178* | Glyco_tranf_GTA_type super family | Constitutive expression | LOC100243459 | Vitis vinifera | 73.26 | 2E－70 |
| Unigene21994 | Glyco_tranf_GTA_type super family | Stem xylem | LOC100248512 | Vitis vinifera | 91.26 | 1E－98 |
| Unigene22477* | PLN02248 | Stem bark | POPTRDRAFT_552489 | Populus trichocarpa | 73.33 | 6E－37 |
| Unigene23146 |  | Stem bark | LOC100781996 | Glycine max | 64.81 | 2E－10 |
| Unigene23587* |  | Stem bark | POPTRDRAFT_1099076 | Populus trichocarpa | 77.5 | 3E－92 |
| Unigene23877 |  | Stem bark | LOC100248512 | Vitis vinifera | 90.12 | 3E－42 |
| Unigene24841 | Glyco_tranf_GTA_type super family | Stem bark | POPTRDRAFT_717644 | Populus trichocarpa | 94.44 | 5E－57 |
| Unigene25822 | Glyco_tranf_GTA_type super family | Stem bark | POPTRDRAFT_553321 | Populus trichocarpa | 44.83 | 8E－15 |
| Unigene26554 |  | Stem bark | LOC100254408 | Vitis vinifera | 41.67 | 1E－11 |
| CL331.Contig1 |  | Leaf | AT5G17420 | Arabidopsis thaliana | 36.93 | 7E－117 |
| Unigene28029 | Glyco_tranf_GTA_type super family | Stem bark | LOC100261696 | Vitis vinifera | 93.07 | 3E－50 |
| Unigene28771 | Glyco_tranf_GTA_type super family | Stem bark | LOC100815436 | Glycine max | 97.48 | 2E－64 |
| Unigene30664* |  | Shoot | AT1G02730 | Arabidopsis thaliana | 60.27 | 6E－21 |
| Unigene30783 | Glyco_tranf_GTA_type super family | Stem bark | LOC100261696 | Vitis vinifera | 84.27 | 3E－42 |
| CL606.Contig1 | Glyco_tranf_GTA_type super family | Stem bark | ARALYDRAFT_487155 | Arabidopsis lyrata | 48.67 | 2E－111 |
| CL686.Contig1 |  | Stem bark | LOC100779834 | Glycine max | 31.13 | 1E－108 |
| CL1504.Contig2* | Glyco_tranf_GTA_type super family | Shoot | LOC100243459 | Vitis vinifera | 92.71 | 7E－67 |
| CL1547.Contig1 |  | Shoot、stem bark | LOC100263604 | Vitis vinifera | 62.03 | 1E－22 |

* mean the gene was assigned into pathway of Starch and sucrose metabolism by KEGG searching
