# Supplementary material for: De novo assembly and characterization of transcriptome using Illumina paired-end sequencing and identification of CesA gene in ramie (Boehmeria nivea L. Gaud)
Source: BMC Genomics. 2013 Feb 26;14:125. doi: 10.1186/1471-2164-14-125 (PMC3610122; doi:10.1186/1471-2164-14-125)
Supplement: Additional file 3: Table S3 — Primer sequence of CesA and 18S RNA genes used for RT-qPCR. [file 1471-2164-14-125-S3.doc]

| Gene | Forward primer (5'-3') | Reverse primer (5'-3') |
| --- | --- | --- |
| CL1504.Contig1 | GGGAGACTCTGACGACGAAG | GAGGCCGGCCATACTTAATC |
| CL1547.contig1 | GAGAGAGAGAGATTTTGTTGGG | GTGGGTGGTGACGAAGAAGT |
| CL3101.Contig2 | ACTTCCATCCCTCACATTGC | AGGTGCTGAGATTGGCTCAC |
| CL331.Contig1 | TTGATTGTTCCACCATGCTC | ATCACCAACTCGCAGCTCTT |
| CL4379.Contig1 | ATGAGCCAACACCCAGAAAC | CCCTGATGATTCCACCAATC |
| CL606.Contig1 | GGCCCTAGAAGACTGTGGTG | TGGGAGTTCACAAAGGCACT |
| CL686.Contig1 | AAACAAGCCCGTTGCTAATG | CCAATCCGATATTCCTCACG |
| Unigene10248 | TTGCTCATTGTTGCAAGGAC | AGGGCACAACGGCATAGTAG |
| Unigene1078 | GTAGCTTGACCGGTGCTCAT | GTTGGGGATCCTGTGAGAGA |
| Unigene1110 | CTGCCTTCGTCCATCTTAGG | CATGGAGGAATGGAGGAAGA |
| Unigene11446 | GCTAAGGCCACTTGGATGTC | GGCTAACATCGCCTGGATAA |
| Unigene11548 | CCTCGCTTACATCAACACCA | AGAACCATATGCTGGCAAGG |
| Unigene11682 | GTGCCCATCTTCGTGGTACT | CTTTTGAGCCGCTTTTTGAC |
| Unigene12047 | CCCATTTGCAAGGACTGTCT | GTGAGAGGCCATTGTGGATT |
| Unigene12173 | GTGGCTCTTTTCAGTTCCCA | GGCTCCAAAATGCTAAACAC |
| Unigene12537 | TACAATGCTGCACCAAATCC | GAAGACAGGCCAAGAGTTCG |
| Unigene12687 | TCTTGATTCCGCCCACTAAG | TCACCGACAAAGTTGTCACC |
| Unigene12727 | GGTTGAATTTCGTGGTACGG | TCGTCGATTTCCTTCTTTGG |
| Unigene1369 | CCTCCAACAACGGTTCTCAT | ACCCAAATGGCAAAGAACAG |
| Unigene14037 | CGGTCGAGATCTTCTTCAGC | GGCAATGAGAGGAAAGGATG |
| Unigene14589 | CTCGTCACTGGAGTCGTCAA | GAGTACCCGATGGTGCTTGT |
| Unigene15308 | AACGAGCAGTTCTGGCTGAT | CAGCGTGAACGAGATGTCC |
| Unigene15343 | GAATCCATGAAAAGGCGAAC | TCATCGCTCGTCTGTTTGTC |
| Unigene16167 | ATAGGCTGGGGTCAGGATTC | GCCAATGTGGAGAAAGATCC |
| Unigene1617 | TGGTTCGCACAGATTGGTTA | GACGCTGACCTCTCGTCTTC |
| Unigene16287 | CATGGAATCAACGCTAGCAA | TCCCACCATTGTTGTCCTTT |
| Unigene16487 | GAGACGAAGACGTCGATGC | CCTTCTCATGGCTTCTCGAC |
| Unigene16621 | AGGCGCATTGGTAAGAACTG | TGCACTGGATGTGGAAGGTA |
| Unigene18262 | GGAGCGTGTAAAAGGATTCAA | GCGGGAAACGTAAACAAGG |
| Unigene18545 | AGCATTGGATTCCGTATTGC | TCGGTCTTCCATGTCCTTGT |
| Unigene18559 | TTGTGCAAGGTCTGCTCAAG | ATAGGGTGGTCGGAGGAATC |
| Unigene19033 | CAACATGACTAGGGCAACCA | GATTTGGTTTGAGGGAAGCA |
| Unigene19910 | GTGTTGATCTCCTCGGTCGT | GTGTCTTCGGGAGGGAAAGT |
| Unigene21178 | TTCCGAAGGCTACTTGGATG | GGAGGTTTCACCATCACCTG |
| Unigene21994 | TCAAGGGTACAGCTCCCATC | AGCTTCCCTTCCTTGTAGCC |
| Unigene22477 | GGAGATCATGGAGACGGAGA | TCCTGGAGCAATCTTCCTTG |
| Unigene2252 | GTACCAAATCGGGCAATGTC | TAAAAGGGCTGCATTCAAGG |
| Unigene23146 | GCTCGGCGTCAGAGACTACT | CTTCCTTCACAACCCTTTCG |
| Unigene23587 | GCTTCTCCACAGGGTACTCG | CCGATCTTCCAGGCATAGAC |
| Unigene23877 | CTTCAAACCTTGTGGGCTTG | CGGATACAGTCCTCCAAAGG |
| Unigene24841 | CAGGGATCACCCTGGTATGA | GGCACCAGCTTTCTTGTGAT |
| Unigene25822 | GCCATCCAATGAGTTCATCA | CCCCATTTTGTTTGACTTCC |
| Unigene26554 | CGCCGTGAAAACGAATACAT | CTCGGAGCTGATTCTCTTCG |
| Unigene28029 | CCTGGGTGTTGGATCAGTTT | TGGATCAACCGTACTCACGA |
| Unigene28771 | CCGATGAAGGAACCTCCTCT | AGCAAACTCTGCGGTTTCTG |
| Unigene306 | CTGGAAGCTCTTCGGCATAC | TTCCTCTGCGAGTCTTGGTT |
| Unigene30664 | TTGAAAACCCGATCACAACC | CTGCTGAGGACGAGGAAGAC |
| Unigene30783 | TTACGGATACAGCCCTGCTT | CACGCTTTGCATCTCGATAG |
| Unigene4349 | CTTTCAAAGGGTCCACGGTA | CTGGATCAGTTCCCCAAATG |
| Unigene5273 | ATGGTTCGGCTTTTACTGGA | TATTGGTGGCTCAATTGTCG |
| Unigene9188 | TCAGACTCCGATGACCTTCC | TCATCGTCCCTTACCTCCTG |
| 18S RNA | ATGATAACTCGACGGATCGC | CTTGGATGTGGTAGCCGT |
